# Targeted Reduction of Specific Antinutritional and Allergen-Associated Proteins to Advance Soybean Seed Quality

**DOI:** 10.3390/ijms27156910

**Published:** 2026-08-01

**Authors:** Hari B. Krishnan, Wonseok Kim, Sunhyung Kim, Thi Thao Nguyen

**Affiliations:** 1 Plant Genetics Research Unit, USDA, Agricultural Research Service, Columbia, MO 65211, USA; 2 Division of Plant Science and Technology, University of Missouri, Columbia, MO 65211, USA; wonseokk@missouri.edu (W.K.); jeongs@missouri.edu (S.K.); 3 Charles W. Gehrke Proteomics Center, Christopher S. Bond Life Sciences Center, University of Missouri, Columbia, MO 65211, USA; tnqp6@missouri.edu

**Keywords:** soybean (*Glycine max* L.), antinutritional factors, allergens, Kunitz trypsin inhibitors, β-conglycinin, seed composition, quantitative proteomics

## Abstract

Soybean (*Glycine max* L.) is a major global source of protein and oil, yet its use in food and feed is constrained by antinutritional factors (ANFs) and allergenic seed proteins. To improve seed nutritional quality, we developed double mutant soybean lines (HBK-WF and HBK-PF) by crossing E16, which lacks the Kunitz trypsin inhibitors KTI-1 and KTI-3, with Lee 2, which is deficient in the α subunit of β conglycinin, a prominent allergen-associated storage protein. One- and two-dimensional gel electrophoresis and immunoblot analyses confirmed the complete absence of these target proteins in both experimental lines. The double mutants exhibited ~50% lower trypsin inhibitor activity relative to wild-type soybean. Tandem mass tag (TMT)-based proteomic analysis quantified 1034 proteins across parental and derived genotypes and revealed extensive proteomic remodeling. Seed composition profiling demonstrated substantial genotypic variation. Lee 2 displayed the highest total protein concentration (44%), while the double mutants exhibited intermediate to elevated levels (39–40%). Oil content in the double mutants (18.4–18.5%) was comparable to E16 and slightly lower than the reference cultivar Maverick. Amino acid profiling further showed that Lee 2 accumulated the highest levels of all measured amino acids, particularly sulfur-containing amino acids, whereas the double mutants displayed intermediate concentrations consistent with additive inheritance. Collectively, these results demonstrate that targeted removal of specific ANFs and an allergen-associated storage protein can improve soybean nutritional quality without compromising seed composition, providing promising germplasm for enhancing the value of soybean-derived food and feed products.

## 1. Introduction

Soybean is the second most economically important crop in the United States after corn, and is valued largely for its high protein and oil content. Commercial soybean seed typically contains roughly 40% protein and 20% oil, making it a major component of animal feed due to its nutritional density, affordability, and broad availability. The predominant storage proteins in soybean seeds are the 7S β-conglycinin and the 11S glycinin fractions [1,2]. β-Conglycinin consists of three subunits (α′, α, and β) with approximate molecular masses of 76, 72, and 53 kDa, while glycinin comprises two major polypeptide groups of 37–44 kDa and 17–22 kDa, encoded by the gy1–gy5 gene family [1,3,4]. In addition to these major storage proteins, soybean seeds accumulate several other protein classes, including lipoxygenases, dehydrins, lectins, and various protease inhibitors [2].

Despite its value as a protein source for humans and monogastric animals, soybean contains several antinutritional factors (ANFs) such as protease inhibitors, phytic acid, lectins, saponins, phytoestrogens, and allergens that can diminish feed efficiency [5,6,7]. Numerous studies have shown that soybean meal can provoke immune responses in monogastric species and impair growth performance in poultry and livestock [8,9,10]. Ingestion of soybean meal has been associated with intestinal inflammation, reduced nutrient absorption, and overall decreased productivity. Several soybean seed proteins are recognized as allergens in food and feed [8,9,10,11,12,13,14]. Among them, β-conglycinin is one of the most potent allergens for both humans and terrestrial livestock [12,13,14,15]. All three of its subunits have been shown to elicit IgE responses in soybean-sensitive individuals [15]. The protein’s resistance to pepsin digestion and its reactivity with IgG in pig plasma further indicate its role as a strong allergen in swine and a likely contributor to intestinal hypersensitivity and inflammation [9,10,15]. The recent advancements in conventional breeding, molecular breeding, and genome editing have enabled the development of hypoallergenic soybeans [16,17,18,19,20].

Another major class of ANFs in soybean is the Kunitz trypsin inhibitors (KTIs), which interfere with digestive proteases and negatively affect animal performance [5,6,7,8]. Raw or insufficiently processed soybean meal can induce pancreatic hypertrophy, liver abnormalities, and hypoglycemia in livestock and poultry [5,8]. Reducing or eliminating KTI activity in soybean seeds would therefore improve the digestibility and nutritional value of soybean meal. Soybeans possess three KTI isoforms, KTI 1, KTI 2, and KTI 3, with KTI 3 being the most abundant. Germplasm screening has revealed useful mutants, including accession PI 157740, which exhibits approximately 40% lower trypsin inhibitor activity due to a frameshift mutation in the KTI-3 coding region [21,22]. This line has been extensively backcrossed and feeding studies have shown that raw extruded meal lacking KTI-3 enhances weight gain relative to conventional soybeans. A second accession, PI 68679, carries a frameshift mutation in KTI-1 [22]. Crossing the KTI-1 mutant with the KTI- 3-deficient line produced a soybean genotype lacking both KTI-1 and KTI-3 proteins, resulting in a substantial reduction in total trypsin inhibitor activity [22].

The U.S. livestock sector remains the primary consumer of soybean meal. Traditionally, soybean meal is heat-treated (“toasted”) to inactivate ANFs before it is incorporated into feed. This process is energy-intensive, increases production costs, and must be carefully controlled to avoid damaging protein quality. Importantly, heat treatment does not neutralize allergenic proteins such as β-conglycinin. Thus, breeding soybean cultivars with inherently low levels of both ANFs and allergens would provide major benefits to the poultry and livestock industries. In this study, we developed experimental soybean lines that are devoid of the α-subunit of β-conglycinin and KTI proteins. These improved lines have the potential to offer greater value to end users and serve as a promising alternative protein source for aquaculture, potentially reducing reliance on fish meal.

## 2. Results

### 2.1. Development of Trypsin Inhibitor and β-Conglycinin α-Subunit Mutant Lines by Conventional Breeding

**Figure 1:**
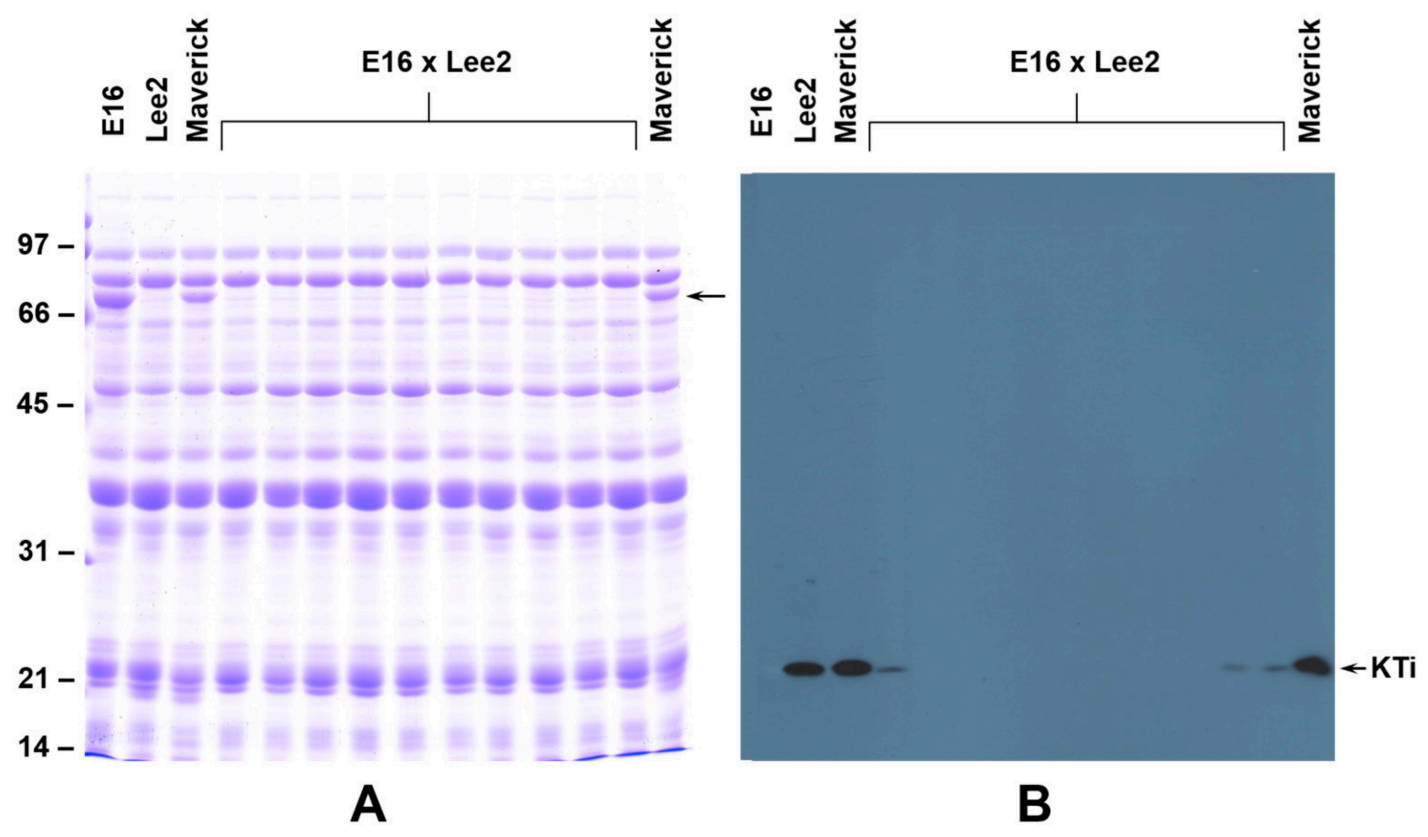
SDS-PAGE analysis of seed proteins and immunological detection of trypsin inhibitor. Total seed proteins extracted from mature dry F_3_ seeds were fractionated in duplicate gels by 10% SDS-PAGE. One gel was stained with Coomassie Blue (**A**), and the other gel was transferred to nitrocellulose membranes. The membrane was probed with the soybean trypsin inhibitor antibodies (**B**). Immunoreactive proteins were identified using anti rabbit IgG-horseradish peroxidase conjugate followed by chemiluminescent. The arrow on the right side of Figure 1A points to the α-subunit of the β-conglycinin. Molecular weight markers in kDa are shown to the left side of the figure. The gels were conducted with two independent biological replications with similar results.

Previously, the identification of a KTI-1 mutant and the stacking of this trait with a KTI-3 mutant were reported [22]. The resulting line, lacking both KTI-1 and KTI-3, was designated E16. This trypsin inhibitor-null line was crossed with Lee 2, a Korean soybean line that lacks the α-subunit of β-conglycinin, a major allergenic protein. F_1_ seeds from these crosses were advanced for three generations by single-seed descent. The introgression of the desired traits, absence of the β-conglycinin α-subunit and absence of KTI, was monitored throughout the breeding process using SDS-PAGE and immunoblot analyses (Figure 1). For comparison, total seed proteins were also isolated from the wild-type soybean cultivar ‘Maverick’ and included in the analysis.

SDS-PAGE analysis of the parental lines and selected F_3_ seeds revealed the expected accumulation of 11S glycinin and 7S β-conglycinin proteins. The α′, α, and β subunits of β-conglycinin, with approximate molecular masses of 76, 72, and 53 kDa, respectively, were detected in E16 and in the wild-type cultivar Maverick. In contrast, the 72 kDa α-subunit was absent in Lee 2 and in all F_3_ seeds derived from the E6 × Lee 2 cross (Figure 1A).

To determine whether these F_3_ seeds also lacked trypsin inhibitor proteins, immunoblot analysis was performed using KTI-specific antibodies. Strong immunoreactivity corresponding to an 18 kDa protein was observed in Lee 2 and Maverick, whereas no signal was detected in E6 (Figure 1B). A faint immunoreactive band was observed in protein extracts from three F_3_ seeds, while the remaining F_3_ seeds showed no detectable reaction with the KTI antibodies (Figure 1B). The faint signals detected in some F3 seeds may be attributed to residual expression or incomplete segregation.

F_3_ lines lacking both the β-conglycinin α-subunit and KTI were selected and advanced to the F_4_ generation. Subsequent self-pollination enabled homozygous fixation of the mutant alleles, resulting in two selected introgressed lines, designated HBK-WF and HBL-PF.

### 2.2. Accumulation of KTI and BBI in HBK-WF and HBK-PF

**Figure 2:**
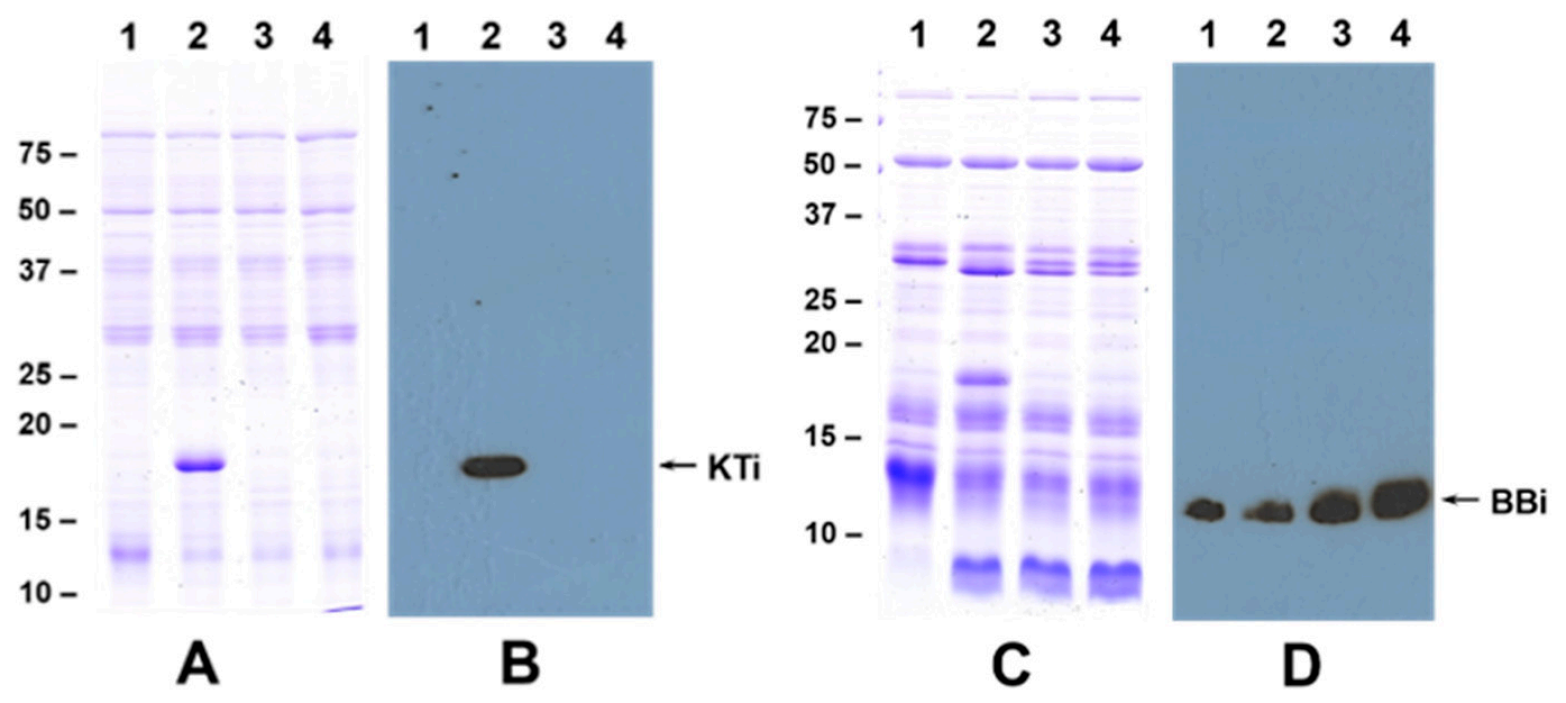
Immunological detection of soybean trypsin and chymotrypsin inhibitors. Calcium fractionated proteins Panel (**A**,**B**) and 50% extracted proteins Panel (**C**,**D**) were fractionated by 13.5% SDS-PAGE. The gels were either stained with Coomassie Blue (**A**,**C**) or transferred to nitrocellulose membranes and probed with either the soybean trypsin inhibitor antibodies (**B**) or soybean Bowman–Birk protease inhibitor antibodies (**D**). Immunoreactive proteins were identified using anti-rabbit IgG-horseradish peroxidase conjugate followed by chemiluminescence. Molecular weight markers in kDa are shown to the left side of the figure. Lane 1—E16; Lane 2—Lee 2; Lane 3—HBK-WF; Lane 4—HBK-PF.

Earlier studies demonstrated that KTI can be preferentially enriched from soybean seed extracts by removing seed storage proteins through cation-mediated precipitation, such as with calcium [23]. Similarly, BBI has been shown to be preferentially extracted using ethanol or isopropanol [24,25]. To assess the presence or absence of KTI in the soybean lines HBK-WF and HBL-PF, a protein fraction was isolated following calcium-induced precipitation of abundant seed storage proteins. Coomassie-stained SDS-PAGE gels revealed a prominent ~18 kDa protein in seed extracts from Lee 2, whereas this band was not observed in extracts from E16, HBK-WF, or HBL-PF (Figure 2A). Immunoblot analysis using antibodies raised against purified soybean KTI-3 confirmed that the abundant protein detected in Lee 2 corresponds to a Kunitz trypsin inhibitor (Figure 2B). No immunoreactive proteins were detected in extracts from E16, HBK-WF, or HBL-PF, indicating that KTI is absent in these lines (Figure 2B).

Trypsin inhibitor activity in soybean is primarily contributed by two proteins: Kunitz trypsin inhibitor (KTI) and Bowman–Birk protease inhibitor (BBI). To determine whether BBI accumulation differed among the experimental soybean lines HBK-WF and HBK-PF, 50% isopropanol-soluble proteins were isolated and analyzed by SDS-PAGE. The Coomassie-stained gel revealed similar overall protein profiles among the four soybean lines examined. However, a prominent low-molecular-weight protein (~4 kDa) was absent in seed extracts of E16 but was clearly present in Lee 2, HBK-WF, and HBK-PF (Figure 2C). Previous studies have identified this protein as leginsulin, a bioactive peptide commonly found in Asian soybean cultivars but less frequently detected in North American cultivars [25]. Western blot analysis using antibodies raised against soybean BBI showed that BBI accumulated in all soybean lines included in the study. Notably, the intensity of the immunoreactive bands was substantially higher in HBK-WF and HBK-PF compared with their parental lines (Figure 2D).

### 2.3. Two-Dimensional Gel Analysis of Total Seed Protein Profiles of Parental and Double-Mutant Experimental Lines

**Figure 3:**
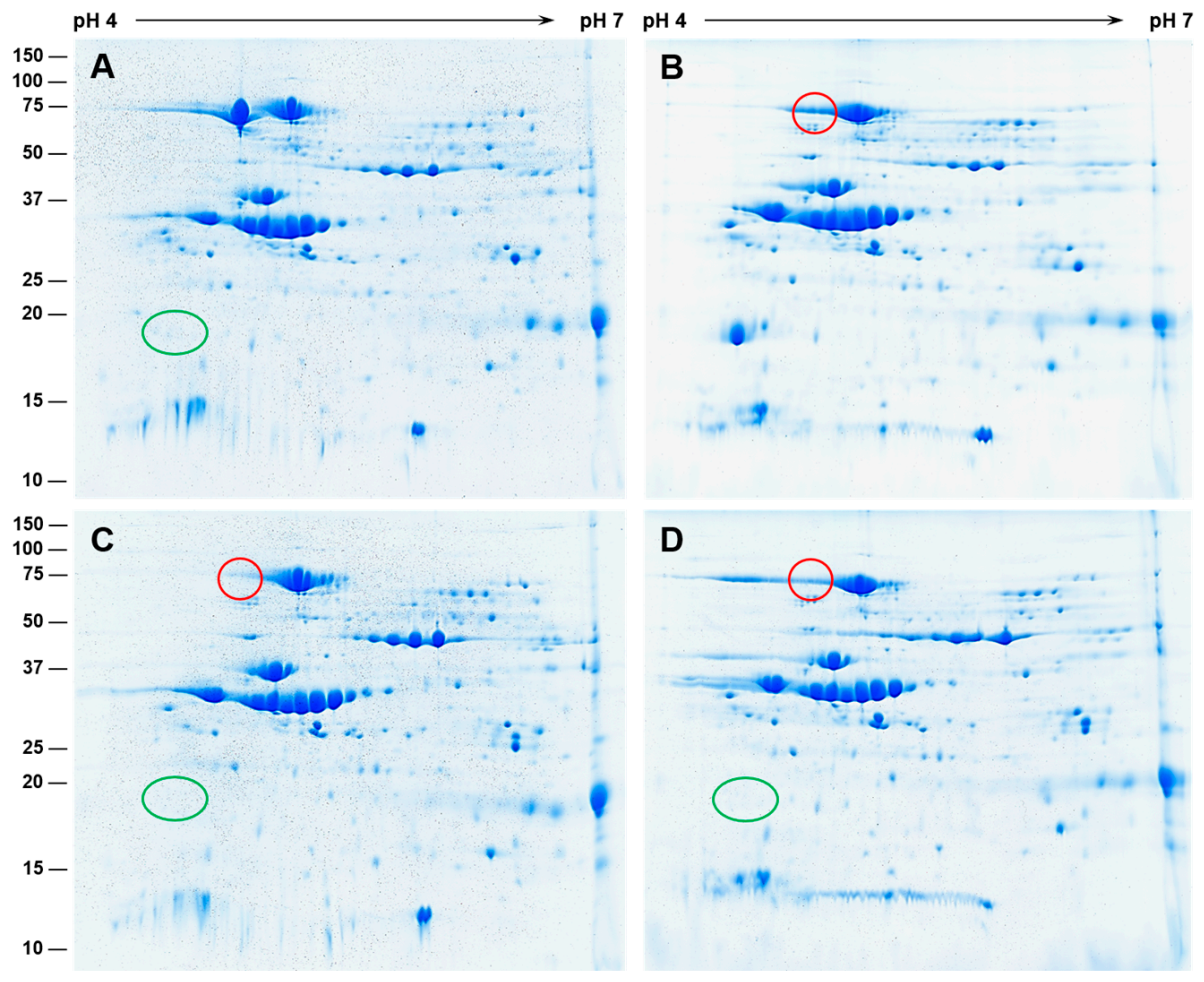
Two-dimensional gel electrophoresis of soybean total seed protein stained with Coomassie brilliant blue. Equivalent amounts of protein (300 μg) were separated on the basis of isoelectric point (pI) and then by 13.5% SDS-PAGE. The position of the α-subunit of the β-conglycinin and KTI proteins is indicated by red and green circles, respectively. Panel (**A**), E16; Panel (**B**), Lee2; Panel (**C**), HBK-WF; Panel (**D**), HBK-PF. Molecular weight markers in kDa are shown to the left side of the figure.

The 2-D gel electrophoretic profiles of the parental and double-mutant soybean lines revealed extensive separation of seed proteins into numerous discrete spots, including the major storage proteins glycinin and β-conglycinin (Figure 3). Analysis of the parental lines, E16 and Lee 2, confirmed the expected absence of the Kunitz trypsin inhibitor (KTI) proteins and the α-subunit of β-conglycinin (Figure 3A,B). In E16, the α′, α, and β subunits of β-conglycinin were readily detected, whereas in Lee 2 the α-subunit was conspicuously missing (Figure 3B). The 18 kDa KTI protein, typically resolved into three closely migrating spots with isoelectric points between pI 4.6 and 4.8, was clearly present in Lee 2 but absent in E16. In the double-mutant experimental lines HBK-WF and HBK-PF, all protein spots corresponding to the α-subunit of β-conglycinin and KTI were absent. This confirms the successful introgression of the mutant alleles into both genetic backgrounds (Figure 3C,D).

### 2.4. Deletion of KTI Proteins Lowers Trypsin Inhibitor Activity but Does Not Eliminate It

**Figure 4:**
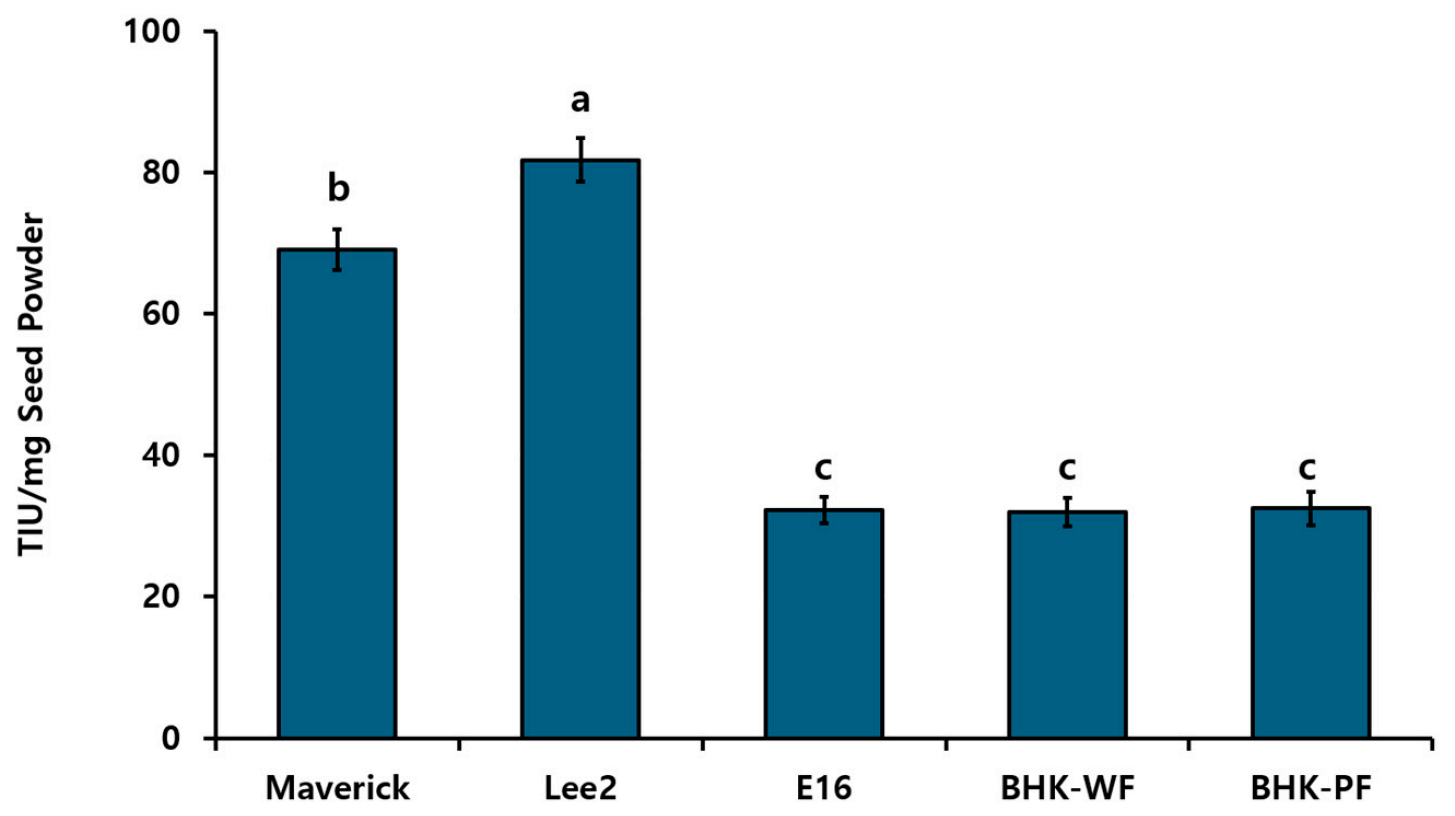
Trypsin inhibitor activity levels present in the seed of selected genotypes. The names of soybean genotypes are indicated below bars. Trypsin inhibitor activity was measured at 37 °C utilizing N-benzoyl-D,L-arginine 4-nitroanilide as substrate. Inhibitor was expressed as TIU (trypsin units inhibited) per milligram seed powder. The error bars indicate the standard error of the mean (*n* = 3). Letters above columns indicate result of one-way ANOVA (*p* < 0.05) and Tukey’s HSD test (α = 0.05); common letters indicate insignificant differences between means.

To evaluate the functional consequences of KTI deletion, trypsin inhibitor activity was quantified in seed extracts. Activity values, expressed as TIU per mg of seed powder, varied substantially among genotypes. Lee 2 exhibited the highest activity at 82 TIU, consistent with its intact KTI loci. The parental line E16, which lacks both KTI-1 and KTI-3, showed approximately 50% lower activity (Figure 4). The homozygous double-mutant lines HBK-WF and HBK-PF displayed activities of 30 TIU and 32 TIU, respectively. For comparison, the reference cultivar Maverick exhibited activity slightly lower than Lee 2.

**Figure 5:**
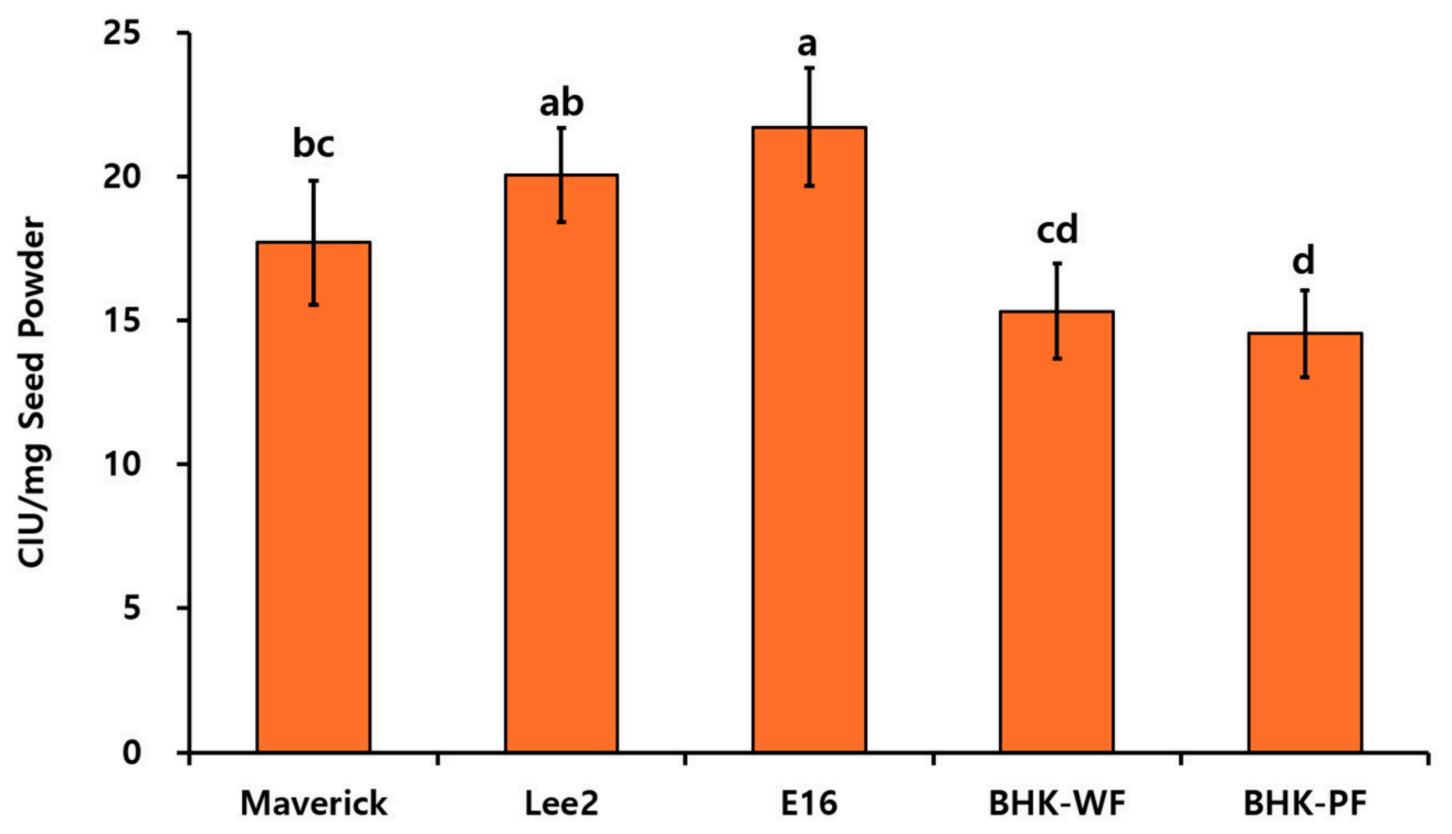
Chymotrypsin inhibitor activity levels present in the seed of selected genotypes. The names of soybean genotypes are indicated below bars. Chymotrypsin inhibitor activity was measured at 37 °C, utilizing N-benzoyl-D,L-arginine 4-nitroanilide as substrate. Inhibitor was expressed as CIU (chymotrypsin units inhibited) per milligram seed powder. The error bars indicate the standard error of the mean (*n* = 3). Letters above columns indicate result of one-way ANOVA (*p* < 0.05) and Tukey’s HSD test (α = 0.05); common letters indicate insignificant differences between means.

These results demonstrate that elimination of KTI-1 and KTI-3 substantially reduces trypsin inhibitor activity but does not completely abolish it. The persistence of roughly 50% residual activity is expected because soybean seeds also accumulate BBI, a protease inhibitor that targets both trypsin and chymotrypsin. The presence of BBI likely accounts for the remaining inhibitory activity in the mutant lines. To evaluate this possibility, chymotrypsin inhibitor activity was also measured from these soybean lines (Figure 5).

In the wild-type cultivar Maverick, chymotrypsin inhibitor activity averaged 17.71 CIU/mg seed powder, whereas the parental lines Lee2 and E-16 exhibited 20.06 and 21.73 CIU/mg seed powder, respectively. Notably, the introgressed lines HBK-WF and HBK-PF showed 15.32 and 14.55 CIU/mg seed powder, values lower than those of the parental genotypes (Figure 5). Although immunoblot analysis revealed a increase in BBI accumulation in the KTI- and β-conglycinin-deficient lines, total chymotrypsin inhibitor activity remained lower than in the parental backgrounds. This outcome underscores that BBI abundance does not directly correspond to functional inhibitory activity, which likely depends on isoform composition, folding efficiency, and the altered protein storage environment resulting from the loss of major seed proteins.

### 2.5. Protein, Oil, and Amino Acid Composition of Parental and Double-Mutant Experimental Lines

The seed composition traits of the parental lines (E16 and Lee 2), the double-mutant experimental lines (HBK-WF and HBL-PF), and the reference cultivar Maverick were also examined. Quantification of protein and oil from seeds harvested from ten individual plants per genotype revealed substantial genotypic variation. Protein content was highest in Lee 2 (44 ± 0.37%), significantly exceeding that of E16 (39 ± 0.35%). The double-mutant lines displayed intermediate to elevated protein levels, HBK-WF averaged 40 ± 1.24%, whereas HBL-PF reached 39 ± 1.39%.

An inverse trend was observed for oil content, consistent with the well-established negative correlation between protein and oil accumulation in soybean. Lee 2 exhibited the lowest oil concentration (16 ± 0.22%), while E16 and Maverick contained 18 ± 0.35% and 20 ± 0.91%, respectively. The double-mutant lines HBK-WF and HBL-PF showed oil contents of 18.50 ± 0.91% and 18.42 ± 0.63%, values comparable to E16 and slightly lower than Maverick.

Analysis of the amino acid profiles revealed that Lee 2 consistently accumulated the highest concentrations of all measured amino acids, reflecting its elevated protein content (Table 1). Notably, the sulfur-containing amino acids, cysteine and methionine, were significantly higher in Lee 2 than in any other genotype. In contrast, Maverick exhibited the lowest levels of these essential amino acids. The double-mutant lines displayed intermediate sulfur amino acid concentrations, consistent with additive contributions from their parental backgrounds (Table 1).

### 2.6. TMT-Based Proteomic Analyses

**Figure 6:**
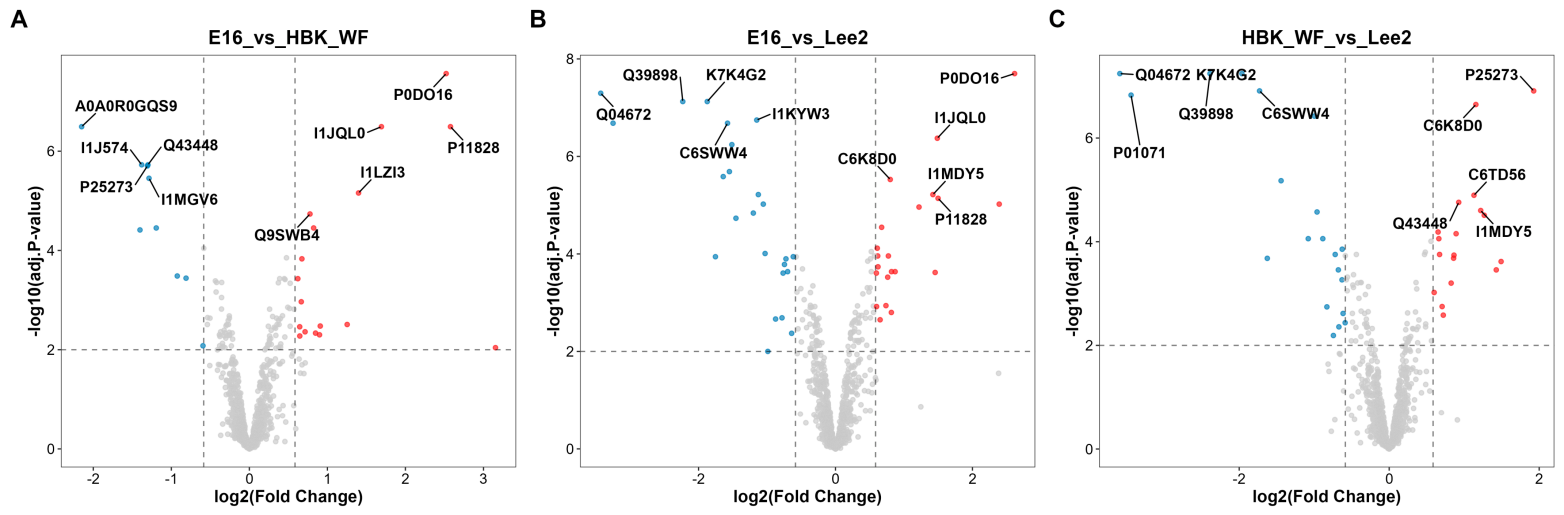
Volcano plots highlighting significance and fold change of differentially expressed proteins (DEPs) in E16 vs. HBK-WF (**A**), E16 vs. Lee2 (**B**), and HBK-WF vs. Lee2 (**C**) comparisons. Red dots represent upregulated proteins, and the blue dots represent downregulated proteins.

Previous studies have demonstrated that removal of highly abundant seed proteins can trigger broad proteomic rebalancing in soybean seeds [26,27]. To determine whether comparable shifts occur in the newly developed experimental soybean line devoid of major allergenic and antinutritional proteins, we conducted a quantitative TMT-based proteomic analysis. Across the three genotypes (Lee2, E16, and HBK-WF), a total of 1034 proteins were confidently quantified. Differential expression analysis identified 79 significantly altered proteins in E16 vs. Lee2, 58 in HBK-WF vs. Lee2, and 79 in E16 vs. HBK-WF (Figure 6 and Supplementary Figures S1–S3).

In the E16 vs. Lee 2 comparison, the α-subunit of β-conglycinin, glycinin-3, a translocator-protein homologue, lysine-tRNA ligase, and Bowman–Birk protease inhibitors were significantly upregulated in E16. Conversely, trypsin inhibitor, sucrose-binding protein, hydrophobic protein, and albumin 1 were downregulated (Figure 6 and Supplementary Table S1). A similar pattern of differential accumulation was observed in the E16 vs. HBK-WF comparison (Figure 6 and Supplementary Table S1). However, in this comparison, albumin 1, cysteine proteinase, hydrophobic seed protein and Kunitz trypsin inhibitor were significantly downregulated in E16 (Figure 6 and Supplementary Table S1). In the HBK-WF vs. Lee2 comparison, trypsin inhibitors KTI-1 and KTI-2 accumulated to higher levels, whereas the most abundant trypsin inhibitor KTI-3, along with sucrose-binding protein and a cupin type-1 domain-containing protein (7S vicilin), showed significant changes in abundance in HBK-WF (Figure 6 and Supplementary Table S1).

**Figure 7:**
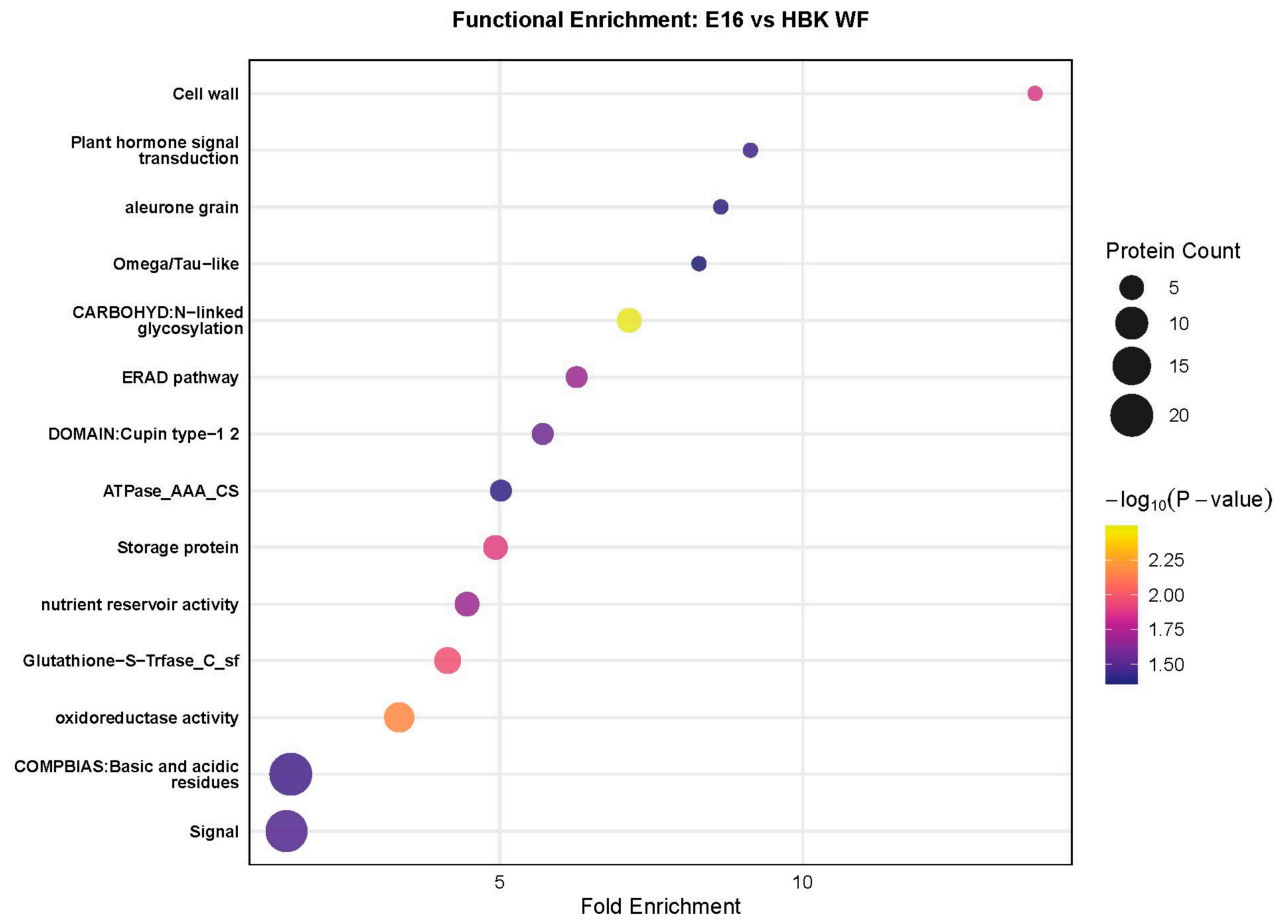
Functional enrichment bubble plots of differentially expressed proteins (DEPs) in E16 vs. HBK-WF comparison. Bubble size indicates the number of proteins assigned to each category.

Functional enrichment analysis revealed that DEPs in the E16 vs. HBK-WF comparison were enriched for pathways related to N-linked glycosylation, oxidoreductase activity, nutrient reservoir activity, signal transduction, and storage-protein biosynthesis (Figure 7). In the HBK-WF vs. Lee2 comparison, enriched categories included Kunitz trypsin inhibitors, other protease inhibitors, proteins associated with hydrogen-peroxide response, signal transduction, and storage-protein biosynthesis (Supplementary Figure S4). DEPs in the E16 vs. Lee2 comparison were distributed across several categories, including storage proteins, cupin-1 family proteins, signaling components, endoplasmic-reticulum–associated proteins, and multiple protease-inhibitor classes (Supplementary Figure S5). Together, these results demonstrate that targeted removal of major allergenic and antinutritional proteins induce a coordinated proteomic rebalancing in the engineered soybean lines, reflecting compensatory adjustments in storage-protein composition, protease-inhibitor networks, and stress-response pathways.

## 3. Discussion

The long-recognized role of trypsin inhibitors as major antinutritional factors in soybean meal has driven decades of research aimed at reducing or eliminating their presence in soybean [6,7]. Their negative impact on protein digestibility and animal growth performance is well established, making the removal of these inhibitors a central objective in soybean improvement. Early efforts identified natural variants with reduced inhibitor activity, such as PI 157740, which carries a ∼40% reduction in trypsin inhibitor levels [21,22]. Subsequent work, including our own identification of PI 68679 with a mutation in KTI-1, expanded the pool of useful alleles [21].

In parallel, plant biotechnology approaches have also been employed to reduce trypsin inhibitor content. Recent applications of CRISPR/Cas9 genome editing have generated transgenic soybean lines with targeted mutations in KTI-1 and KTI-3 [28]. However, despite these advances, all such lines retain significant residual trypsin inhibitor activity due to the continued presence of Bowman–Birk inhibitor (BBI) proteins in the seed. Previously, it was reported that the elimination of KTI-1 and KTI-3 resulted in increased chymotrypsin inhibitor activity, presumably due to elevated accumulation of BBI in this mutant line [22].

In KTI-deficient backgrounds, BBI often increases through compensatory regulation, yet this rise does not improve nutritional quality and instead preserves the overall protease-inhibitor load, leaving residual BBI that still must be thermally inactivated before the meal can be used in monogastric feed. Recent gene-silencing efforts using CRISPR/Cas9 and RNA interference have produced soybean lines with markedly reduced inhibitor activity [28,29,30,31], but remaining inhibitor levels still necessitate heat treatment before inclusion in animal feed. Meaningful improvement of soybean meal quality requires reducing total protease-inhibitor activity, including both KTI and BBI. Eliminating both inhibitor families is essential for maximizing protein digestibility and enabling soybean meal use in monogastric feed formulations without additional thermal processing.

Soybeans constitute a major source of high-quality protein for both human consumption and livestock nutrition. Nevertheless, several soybean proteins are recognized as clinically relevant food allergens, particularly in infants and young children [32]. According to the International Union of Immunological Societies, 18 soybean allergens have been identified to date, including β conglycinin, glycinin, lectin, P34, and Kunitz trypsin inhibitor [33]. Among these, β conglycinin has been repeatedly implicated as a principal allergen in monogastric animals. This storage protein is composed of three subunits, α′ (72 kDa), α (70 kDa), and β (52 kDa), each of which exhibits allergenic properties [15,33]. Evidence indicates that the α subunit is the predominant immunoreactive component responsible for eliciting allergic responses in young pigs [13,15]. In susceptible piglets, β conglycinin exposure commonly results in digestive disturbances, growth retardation, and diarrhea, reflecting an underlying hypersensitivity reaction. Recent studies further demonstrate that β conglycinin can compromise intestinal barrier integrity in weaned piglets, inducing oxidative stress and inflammatory signaling pathways [33,34].

Given the well-documented adverse physiological effects of β-conglycinin on young monogastric animals, reducing or eliminating this storage protein from soybean-derived feed ingredients remains a promising strategy for improving feed safety and nutritional quality. Several studies have reported naturally occurring or radiation-induced mutants affecting one or more β-conglycinin subunits, as well as biotechnological approaches that generate soybean lines completely lacking this protein [35,36,37,38]. In the present study, we generated soybean lines that lack not only the abundant Kunitz trypsin inhibitor but also the α-subunit of β-conglycinin, a dominant allergen. These modified soybean lines represent a promising protein source for monogastric animals, potentially improving digestibility and reducing allergenic responses. To determine their practical value, comprehensive feeding trials are needed to assess both the nutritional performance and safety of these modified soybean proteins in young monogastric animals.

Soybean genotype Lee2 exhibited a notably higher protein content, aligning with previous findings in the HP1 mutant of GmCG-1, which encodes the β-conglycinin α′ subunit, which also demonstrated increased protein concentration and enhanced amino acid digestibility [39]. Our results further show that Lee 2 possessed a superior amino acid profile, reinforcing the hypothesis that the absence of β-conglycinin may be mechanistically linked to improvements in both protein content and protein quality. This interpretation is supported by additional work demonstrating that knockdown of the β-conglycinin α and α′ subunits not only enhance seed nutritional quality but also confers increased salt tolerance in soybean plants [40]. Taken together, these findings highlight the broader potential of β-conglycinin-deficient mutants as valuable genetic resources for developing soybean varieties with improved nutritional attributes and enhanced abiotic stress tolerance.

Global demand for high-quality protein for both human food and animal feed continues to increase, yet modern soybean breeding has largely emphasized seed yield at the expense of seed composition. This emphasis has contributed to a long-recognized negative correlation between yield and protein concentration, leading to a progressive decline in soybean protein content over several decades [20,41]. Within this context, the newly developed experimental lines represent a significant advancement. They simultaneously reduce allergenic and antinutritional factors and reverse the downward trajectory of seed protein concentration, thereby improving both the nutritional quality and functional value of soybean. These lines provide a promising genetic resource for future breeding efforts aimed at enhancing soybean protein content and quality. Although the newly developed soybean lines successfully eliminate KTI and the β-conglycinin α-subunit, the study did not include allergenicity testing to determine whether these modifications meaningfully reduce immunoreactivity in sensitive monogastric animals. Likewise, no feeding experiments were conducted, leaving unresolved questions about digestibility, growth performance, gut physiology, and overall safety under real-world dietary conditions. Finally, the lack of field performance evaluation means that agronomic stability, yield potential, and environmental responsiveness of these experimental lines remain unknown. Collectively, these gaps underscore the need for comprehensive immunological, nutritional, and agronomic validation before the full utility of these modified soybean genotypes can be established.

## 4. Materials and Methods

### 4.1. Plant Materials

The soybean line E16, which lacks both KTI-1 and KTI-3 isoforms of the Kunitz trypsin inhibitor, has been described previously [22]. E16 was developed through traditional hybridization by crossing a line carrying the KTI-1^−^ mutation (PI 68679) with a line carrying the previously identified KTI-3^−^ mutation (PI 542044). The Korean soybean cultivar Lee 2, which naturally lacks the α-subunit of β-conglycinin, was generously provided by Dr. Byongwon Lee of the National Institute of Crop Science, RDA, Korea.

### 4.2. Development of Soybean Lines Lacking Kunitz Trypsin Inhibitors (KTi-1/KTi-3) and the α-Subunit of β-Conglycinin

The two parental lines (E16 and Lee2) were used in a conventional breeding program to develop experimental soybean lines simultaneously lacking KTI-1, KTI-3, and the α-subunit of β-conglycinin. Reciprocal crosses were performed to ensure that maternal effects were accounted for in the breeding process. In one cross, E16 served as the female parent (E16 × Lee 2), while in the reciprocal cross, Lee 2 served as the female parent (Lee 2 × E16). F_1_ plants from both directions were self-pollinated to produce segregating F_2_ seed populations. To identify individuals carrying the desired trait combination, seed chips from individual F_2_ seeds were analyzed for the absence of KTI proteins and the α-subunit of β-conglycinin using SDS-PAGE followed by immunoblotting. F_2_ seeds lacking all three target proteins were selected and advanced through subsequent generations. Line advancement continued until homozygosity for the absence of KTI-1, KTI-3, and the α-subunit of β-conglycinin was confirmed. Ultimately, two homozygous lines (HBK-WF and HBK-PF), one derived from each reciprocal cross, were selected. These lines were grown under field conditions at the University of Missouri Bradford Research Farm for further evaluation.

### 4.3. Protein Isolation and SDS-PAGE Analysis

Dry soybean seeds were ground into a fine powder, and 10 mg of the homogenized material was placed into a 2 mL microcentrifuge tube. Protein extraction was carried out by adding 1 mL of SDS sample buffer (0.06 M Tris-HCl, pH 6.8, containing 2% SDS, 10% glycerol, and 5% 2-mercaptoethanol). The mixture was vortexed vigorously for 10 min at room temperature, then heated at 100 °C for 5 min to ensure complete solubilization. Samples were centrifuged at 15,800× *g* for 5 min, and the clarified supernatant was transferred to new tubes for analysis.

Protein separation was conducted using a Hoeffer SE250 Mini Vertical SDS-PAGE apparatus (GE Healthcare, Pittsburgh, PA, USA). Approximately 50 µg of total seed protein was loaded onto a 13.5% acrylamide gel, and electrophoresis proceeded at a constant current of 20 mA per gel until the tracking dye reached the lower edge. Following electrophoresis, gels were removed and stained overnight with Coomassie Brilliant Blue R-250 (Sigma-Aldrich, St. Louis, MO, USA).

### 4.4. Two-Dimensional SDS-PAGE

Proteins were isolated from 200 mg of finely ground soybean seed powder using 5 mL of ice-cold 0.1 M Tris-Cl (pH 8.8) containing 0.9 M sucrose, 0.25 M NaCl, 0.4% (*w*/*v*) β-mercaptoethanol, and a Plant ProteaseArrest inhibitor cocktail (G-Biosciences, St. Louis, MO, USA). The suspension was vortexed thoroughly and kept on ice for 15 min. An equal volume of saturated phenol (pH 4.3) was added, and the mixture was continuously agitated for 30 min at 25 °C. After centrifugation at 5000× *g* for 20 min at 23 °C in a swing-bucket rotor, the upper phenolic phase was collected and mixed with ten volumes of cold methanol containing 0.1 M ammonium acetate. Proteins were precipitated by incubating the mixture at −80 °C for 2 h.

The protein pellet was recovered by centrifugation at 12,000× *g* for 15 min at 4 °C, washed twice with methanol containing 0.1 M ammonium acetate and 0.01 M DTT, and washed three additional times with acetone containing 0.01 M DTT. After briefly air-drying, the pellet was dissolved in an IEF buffer composed of 7 M urea, 2 M thiourea, 1% (*w*/*v*) CHAPS, and 2% (*w*/*v*) C7BzO.

For isoelectric focusing, 300 µg of protein was applied to each strip. Linear 13 cm IPG strips (pH 4–7) were rehydrated to 250 µL in IEF buffer containing the sample and adjusted to final concentrations of 5% (*v*/*v*) glycerol, 2.2% (*v*/*v*) 2-HED, and 0.06 M DTT. Strips were passively rehydrated and focused using a Protean II system (Bio-Rad, Hercules, CA, USA). After focusing, strips were equilibrated for 10 min in a urea-based buffer (0.05 M Tris-Cl, pH 8.8, 6 M urea, 30% glycerol, 5% SDS, 0.01% bromophenol blue) containing 2% (*w*/*v*) DTT, followed by a second 10 min equilibration in the same buffer containing 2.5% (*w*/*v*) iodoacetamide.

Proteins were separated in the second dimension on a 12–16% T gradient SDS-PAGE gel. Electrophoresis was carried out on a SE600 system (Hoefer Inc., Bridgewater, MA, USA) at 20 mA per gel for 45 min, then increased to 50 mA per gel until completion. Gels were fixed for 30 min in a 5:4:1 methanol:water:acetic acid solution, rinsed twice with ultrapure water, and stained overnight with Coomassie G-250 (Sigma-Aldrich, St. Louis, MO, USA). One- and two-dimensional gels were destained with repeated water washes and scanned individually using an Epson V700 (Los Alamitos, CA, USA) Perfection scanner operated through Adobe Photoshop (Version 2016).

### 4.5. Immunoblot Analysis

Proteins resolved by SDS–PAGE were transferred onto 0.45 µm nitrocellulose membranes using the method described previously [15]. Membranes were then incubated for 1 h at 25 °C in Tris-buffered saline (TBS; pH 7.3) supplemented with 5% nonfat dry milk to block nonspecific binding. Each membrane was exposed overnight at 25 °C with gentle agitation to primary antibodies diluted 1:10,000, targeting soybean trypsin inhibitor and the soybean Bowman–Birk protease inhibitor. The antibodies were raised in rabbits against purified soybean KTI and BBI proteins [29,30]. Following primary incubation, membranes were washed four times for 10 min each in TBS containing 0.05% Tween-20 (TBST). Detection was carried out using a horseradish peroxidase–conjugated goat anti-rabbit IgG secondary antibody (1:5000; Bio-Rad, Hercules, CA, USA) for 2 h. Immunoreactive protein bands were visualized using the SuperSignal West Pico chemiluminescent substrate (Pierce, Rockford, IL, USA) according to the manufacturer’s protocol.

### 4.6. Seed Amino Acid Analysis

Total amino acid levels were determined after subjecting soybean seed powder to acid hydrolysis. Samples were placed in sealed, evacuated tubes containing 6 N HCl and incubated at 110 °C for 22 h. Following hydrolysis, amino acid profiles were measured using a Hitachi L-8000 amino acid analyzer (Hitachi, Tokyo, Japan). Because sulfur-containing residues degrade under standard hydrolysis conditions, methionine and cysteine were quantified separately from duplicate aliquots that were first oxidized with performic acid and then hydrolyzed in 6 N HCl. All measurements were carried out in triplicate, and the resulting data were subjected to appropriate statistical analyses.

### 4.7. Trypsin and Chymotrypsin Inhibitor Assay

Trypsin inhibitor activity in the soybean samples was assessed following the procedure outlined by the referenced method [42]. Briefly, 20 mg of finely ground seed material was placed in a 2 mL microcentrifuge tube and extracted with 1 mL of 10 mM NaOH. The mixture was vigorously vortexed for 10 min at room temperature and then centrifuged at 16,000× *g* for 10 min to obtain a clarified extract. The resulting supernatant served as the source material for determining KTI activity. Trypsin inhibition was quantified using a reaction mixture containing 200 μL of assay buffer (50 mM Tris-HCl, pH 8.2, supplemented with 20 mM CaCl_2_), 500 μL of Nα-benzoyl-DL-arginine 4-nitroanilide hydrochloride (0.4 mg/mL), and 200 μL of trypsin (20 μg/mL). Reactions were incubated at 37 °C for 10 min and terminated by the addition of 200 μL of 30% acetic acid. Absorbance was measured at 410 nm, and trypsin inhibition was expressed as trypsin units inhibited per milligram of sample (TIU/mg).

Chymotrypsin inhibitor activity in soybean seeds was measured following the procedure described earlier [30] utilizing N-Succinyl-Ala-Ala-Pro-Phe p-nitroanilide (AAPF) as the substrate. The results are presented as the mean ± SD from three biological replicates. Data were statistically evaluated using JMP 16.0 (SAS Institute, Cary, NC, USA). A one-way ANOVA was performed, and significant differences among means (α = 0.05) were identified using the Tukey–Kramer HSD test.

### 4.8. TMT-Based Proteomic Analyses

Protein extraction from soybean seeds was carried out using three biological replicates and adhered to the protocol established for 2-D gel electrophoresis. For each soybean line, 100 µg of protein extract was reduced with DTT, alkylated with iodoacetamide, and digested overnight at 37 °C using trypsin/Lys-C. Digestion was quenched with formic acid, and samples were dried and desalted using a BioPureSPF Easy Flo PROTO 300 C18 Midi plate (Thermo Fisher Scientific, Waltham, MA, USA) before final drying.

For TMT labeling, samples were reconstituted in 100 µL of TEAB, quantified by BCA assay, and 25 µg of each sample was labeled with TMT10plex reagents, and the pooled reference was labeled with TMT11 (131C). For LC-MS/MS, 1 µg of each labeled sample plus 1 µg of reference were combined, dried, and reconstituted in 5% acetonitrile/0.1% formic acid. A 5 µg aliquot was injected for analysis. The peptides were then separated by liquid chromatography (Thermo Scientific nLC-1200) and introduced to the mass spectrometer (Thermo Scientific Q-Exactive) using a Thermo Scientific Nanospray FlexIon source. The peptide ions were subjected to HCD fragmentation and reporter ions generated from the TMTs were used for quantitative analysis.

Raw data were processed using Thermo Scientific Proteome Discoverer 3.0. Peptide-spectrum matches were assigned by comparing observed intact and fragment ion patterns with theoretical spectra generated by Mascot (Matrix Science, London, UK) or Sequest HT (Thermo Fisher Scientific, Waltham, MA, USA). Database searches were performed with static modifications of carbamidomethyl (Cys) and TMT label (Lys and N-termini) and dynamic modifications of oxidation (Met) and deamidation (Asn,Gln).

Relative protein abundances were calculated as ratios normalized to the 131C reference channel. Missing values were imputed during Proteome Discoverer preprocessing. Pre-imputed abundance ratios for E16, Lee2, and HBK-WF (biological triplicates) were imported into R for downstream analysis using the DEP (version 1.12.0) and SummarizedExperiment packages (version 1.42.0). Variance-stabilizing normalization (VSN) was applied prior to statistical testing.

Pairwise differential expression analyses (E16 vs. Lee2, HBK-WF vs. Lee2, E16 vs. HBK-WF) were performed using empirical Bayes statistics within DEP. Proteins were considered significantly differentially expressed at FDR < 0.05 and absolute log_2_FC ≥ 0.5. All figures were generated in R (version 4.5.3).

### 4.9. GO Enrichment Analysis

Differentially abundant or strain-specific proteins identified from the pairwise comparisons (E16 vs. Lee2, HBK-WF vs. Lee2, and E16 vs. HBK-WF) were subjected to functional enrichment analysis using DAVID (v2026). To reduce statistical bias common in mass spectrometry-based enrichment studies, the background set was defined as all proteins detected across the full experiment. Functional over-representation was assessed using the DAVID EASE score (modified Fisher’s exact test), applying significance thresholds of *p* < 0.05 and a minimum term size of ≥2 proteins. Annotation categories included Gene Ontology Biological Process, Molecular Function, and Cellular Component terms, as well as InterPro and SMART domains, UniProt keywords, and sequence features.

Redundant annotation terms with identical gene counts and statistical values were collapsed into representative entries to streamline interpretation. Significantly enriched terms were visualized in R using ggplot2 (version 4.0.0) as bubble plots summarizing enrichment significance and functional category representation. All visualizations were generated automatically from DAVID output tables using custom R scripts (version 4.5.3).

## Figures and Tables

**Table 1:** High-performance liquid chromatography measurement of amino acid content of soybean seed proteins (% per 100 g protein).

Amino acid	Lee 2	E16	BHK-WF	BHK-PF	Maverick
	(Mean ± SD)	(Mean ± SD)	(Mean ± SD)	(Mean ± SD)	(Mean ± SD)
Aspartic Acid	5.11 ± 0.01 ^a^	4.46 ± 0.05 ^c^	4.62 ± 0.05 ^b^	4.37 ± 0.07 ^d^	4.16 ± 0.01 ^e^
Threonine	1.82 ± 0.02 ^a^	1.52 ± 0.03 ^d^	1.65 ± 0.01 ^b^	1.58 ± 0.02 ^c^	1.51 ± 0.01 ^d^
Serine	1.94 ± 0.08 ^a^	1.74 ± 0.06 ^c^	1.89 ± 0.01 ^ab^	1.81 ± 0.01 ^abc^	1.74 ± 0.03 ^bc^
Glutamic Acid	7.80 ± 0.18 ^a^	7.24 ± 0.08 ^b^	7.29 ± 0.07 ^b^	6.86 ± 0.10 ^c^	6.39 ± 0.06 ^d^
Proline	2.23 ± 0.03 ^a^	1.99 ± 0.01 ^b^	2.02 ± 0.02 ^b^	1.92 ± 0.03 ^bc^	1.79 ± 0.03 ^c^
Glycine	2.02 ± 0.00 ^a^	1.66 ± 0.01 ^d^	1.85 ± 0.02 ^b^	1.75 ± 0.01 ^c^	1.69 ± 0.02 ^d^
Alanine	1.93 ± 0.01 ^a^	1.70 ± 0.02 ^c^	1.77 ± 0.02 ^b^	1.71 ± 0.02 ^c^	1.62 ± 0.01 ^d^
Cysteine	0.79 ± 0.00 ^a^	0.66 ± 0.02 ^cd^	0.69 ± 0.01 ^bc^	0.70 ± 0.02 ^b^	0.61 ± 0.01 ^d^
Valine	2.33 ± 0.06 ^a^	1.99 ± 0.01 ^bc^	2.14 ± 0.03 ^b^	2.05 ± 0.03 ^b^	1.90 ± 0.02 ^c^
Methionine	0.64 ± 0.00 ^a^	0.57 ± 0.01 ^b^	0.58 ± 0.01 ^b^	0.56 ± 0.01 ^b^	0.53 ± 0.00 ^c^
Isoleucine	2.13 ± 0.03 ^a^	1.94 ± 0.01 ^b^	1.86 ± 0.02 ^c^	1.78 ± 0.02 ^d^	1.71 ± 0.0a ^e^
Leucine	3.33 ± 0.01 ^a^	3.04 ± 0.04 ^b^	3.05 ± 0.03 ^b^	2.93 ± 0.04 ^c^	2.80 ± 0.02 ^d^
Tyrosine	1.71 ± 0.05 ^a^	1.52 ± 0.04 ^b^	1.53 ± 0.01 ^b^	1.48 ± 0.01 ^b^	1.39 ± 0.02 ^c^
Phenylalanine	2.29 ± 0.02 ^a^	2.07 ± 0.04 ^b^	2.03 ± 0.02 ^bc^	1.97 ± 0.02 ^c^	1.88 ± 0.02 ^d^
Lysine	2.94 ± 0.00 ^a^	2.60 ± 0.03 ^b^	2.65 ± 0.02 ^b^	2.54 ± 0.02 ^c^	2.45 ± 0.01 ^d^
Histidine	1.29 ± 0.01 ^a^	1.06 ± 0.01 ^d^	1.17 ± 0.01 ^b^	1.11 ± 0.01 ^c^	1.00 ± 0.01 ^e^
Arginine	3.68 ± 0.04 ^a^	2.84 ± 0.04 ^c^	2.96 ± 0.02 ^b^	2.91 ± 0.05 ^bc^	2.56 ± 0.02 ^d^

Values represent the mean ± standard error from three biological replicates. Lowercase letters indicate statistical groupings based on one-way ANOVA (*p* < 0.05) followed by Tukey’s HSD (α = 0.05); different letters denote significant differences among genotype means.

## Data Availability

The original contributions presented in this study are included in the article and Supplementary Materials. Further inquiries can be directed to the corresponding author.

## Supplementary Materials

The following supporting information can be downloaded at https://www.mdpi.com/article/10.3390/ijms27156910/s1.

## Abbreviations

The following abbreviations are used in this manuscript:

ANF	Antinutritional Factors
BBI	Bowman–Birk Protease Inhibitor
KTI	Kunitz Trypsin Inhibitor
TMT	Tandem Mass Tag
DEPs	Differentially Expressed Proteins
FC	Fold Change
