# Supplementary material for: Targeted Reduction of Specific Antinutritional and Allergen-Associated Proteins to Advance Soybean Seed Quality
Source: Int J Mol Sci. 2026 Aug 1;27(15):6910. doi: 10.3390/ijms27156910 (PMC13467562; doi:10.3390/ijms27156910)
Supplement: Supplementary file 1 [file ijms-27-06910-s001.zip › ijms-4445254-supplementary.pdf]

Supplemental Materials

Supplemental Figure S1. Heatmap showing differential protein accumulation in E16 and Lee2.

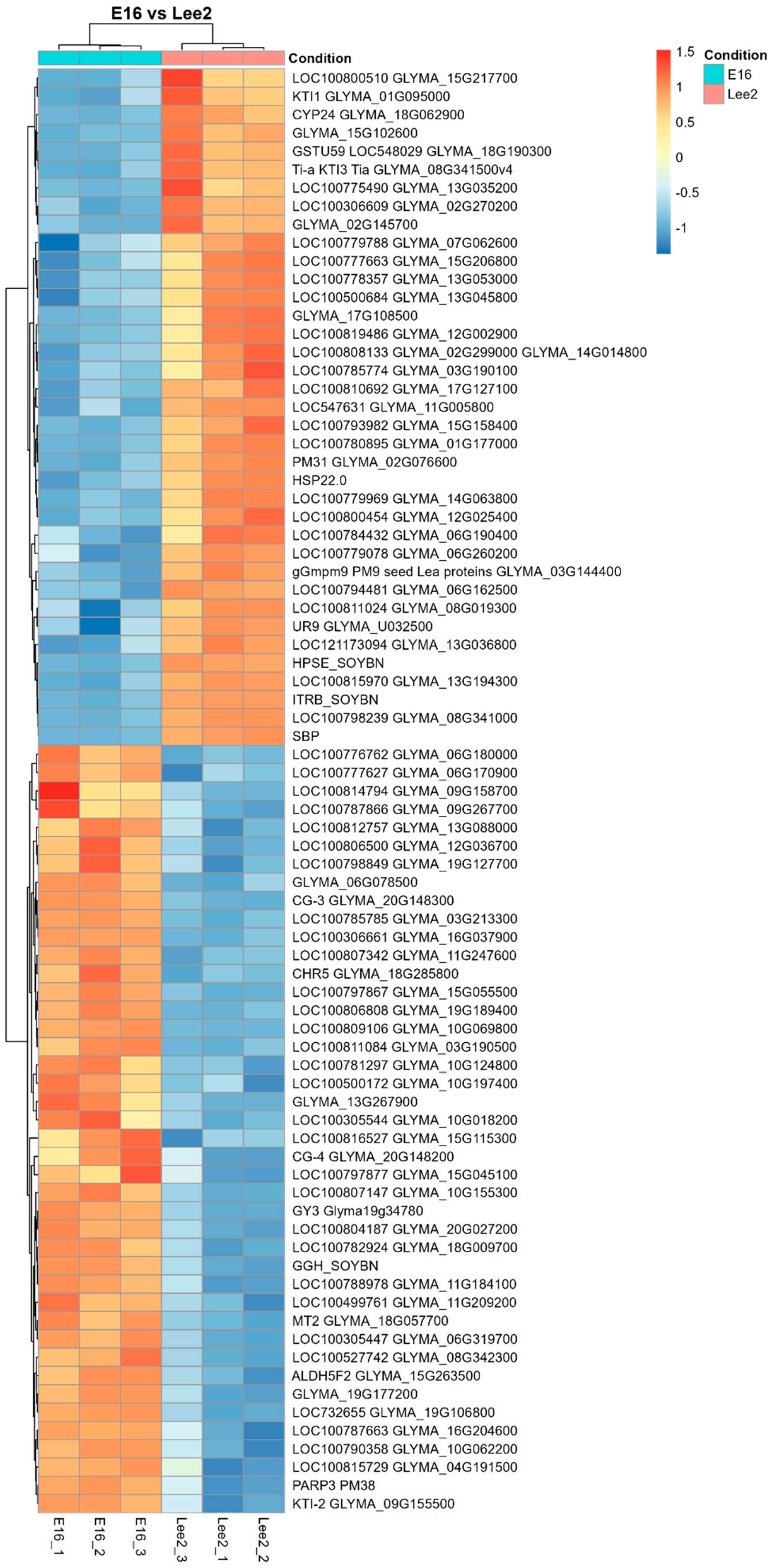

Supplemental Figure S2. Heatmap showing differential protein accumulation in HBK-WF and Lee2.

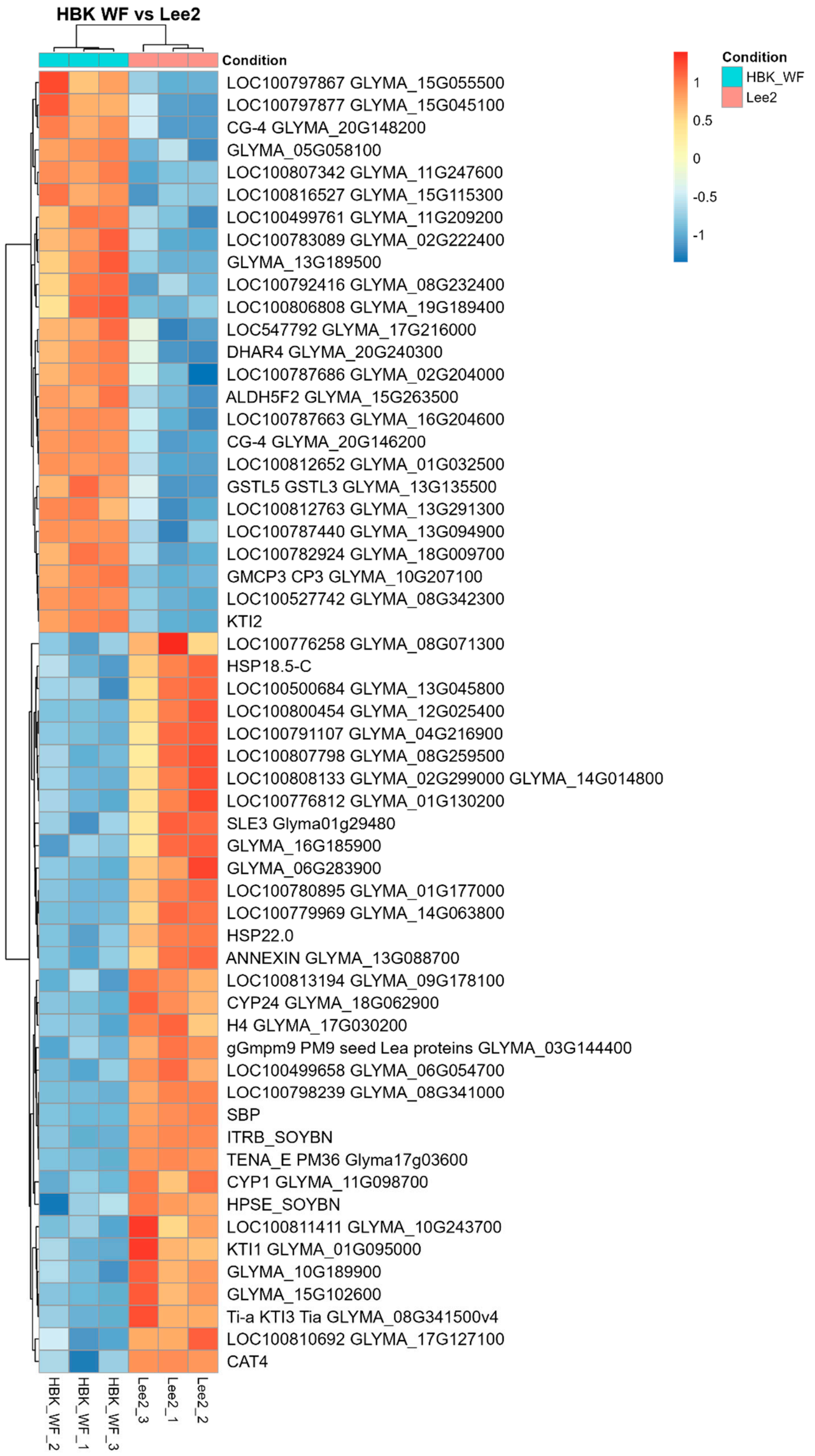

Supplemental Figure S3. Heatmap showing differential protein accumulation E16 and HBK-WF.

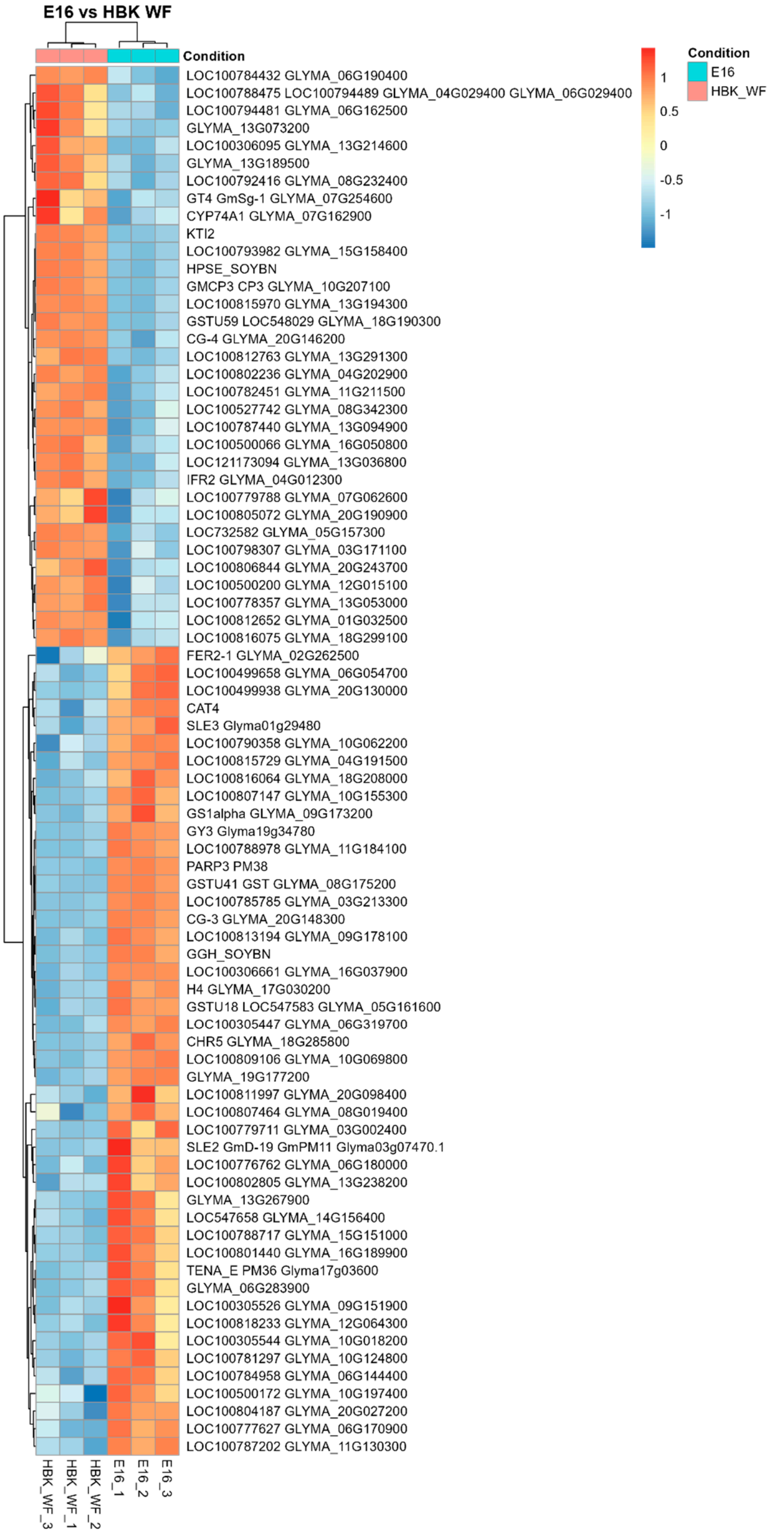

Supplemental Figure S4: Functional enrichment bubble plots of differentially expressed proteins (DEPs) in HBK-WF and Lee2 comparison.

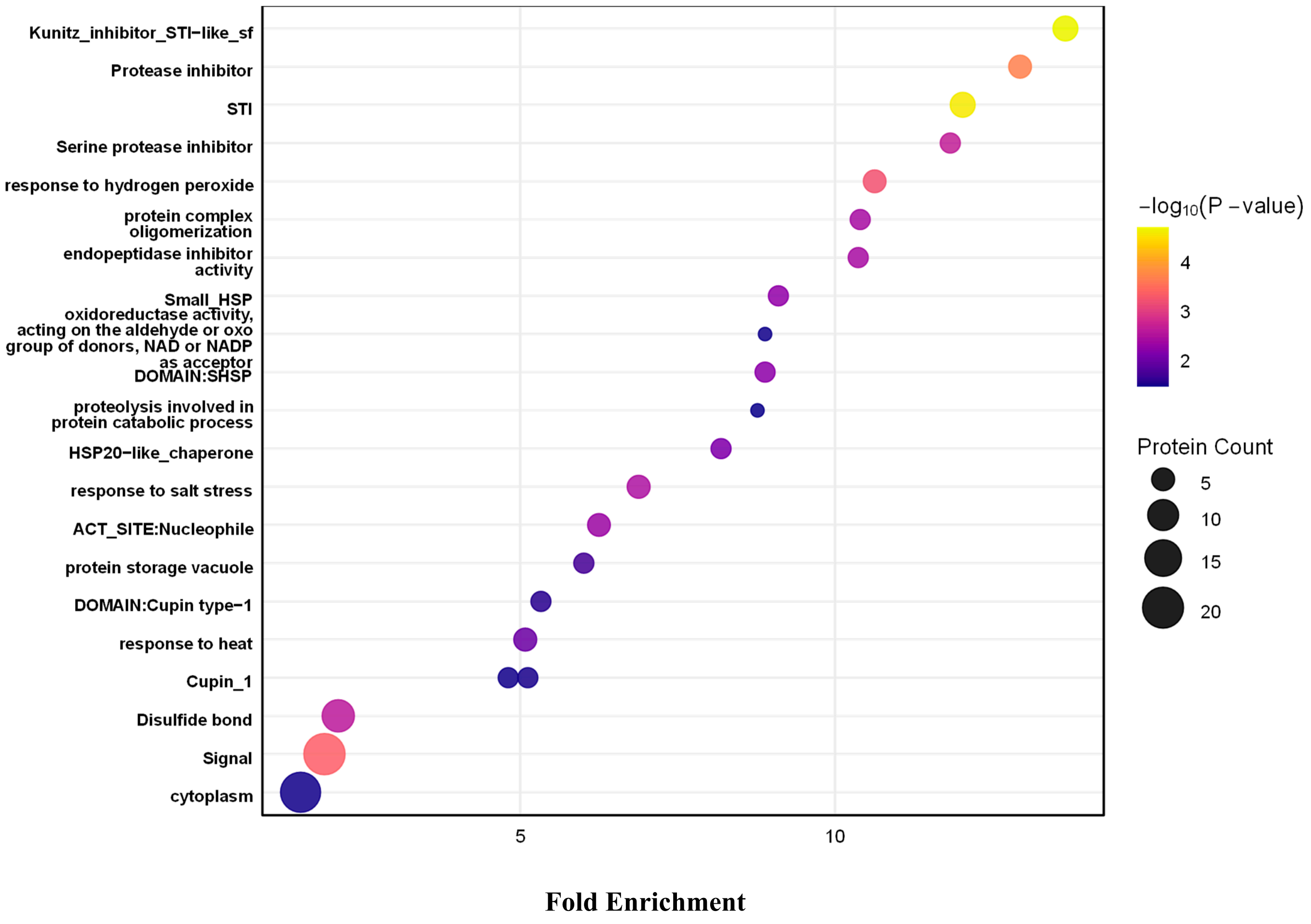

Supplemental Figure S5: Functional enrichment bubble plots of differentially expressed proteins (DEPs) in E16 and Lee2 comparison.

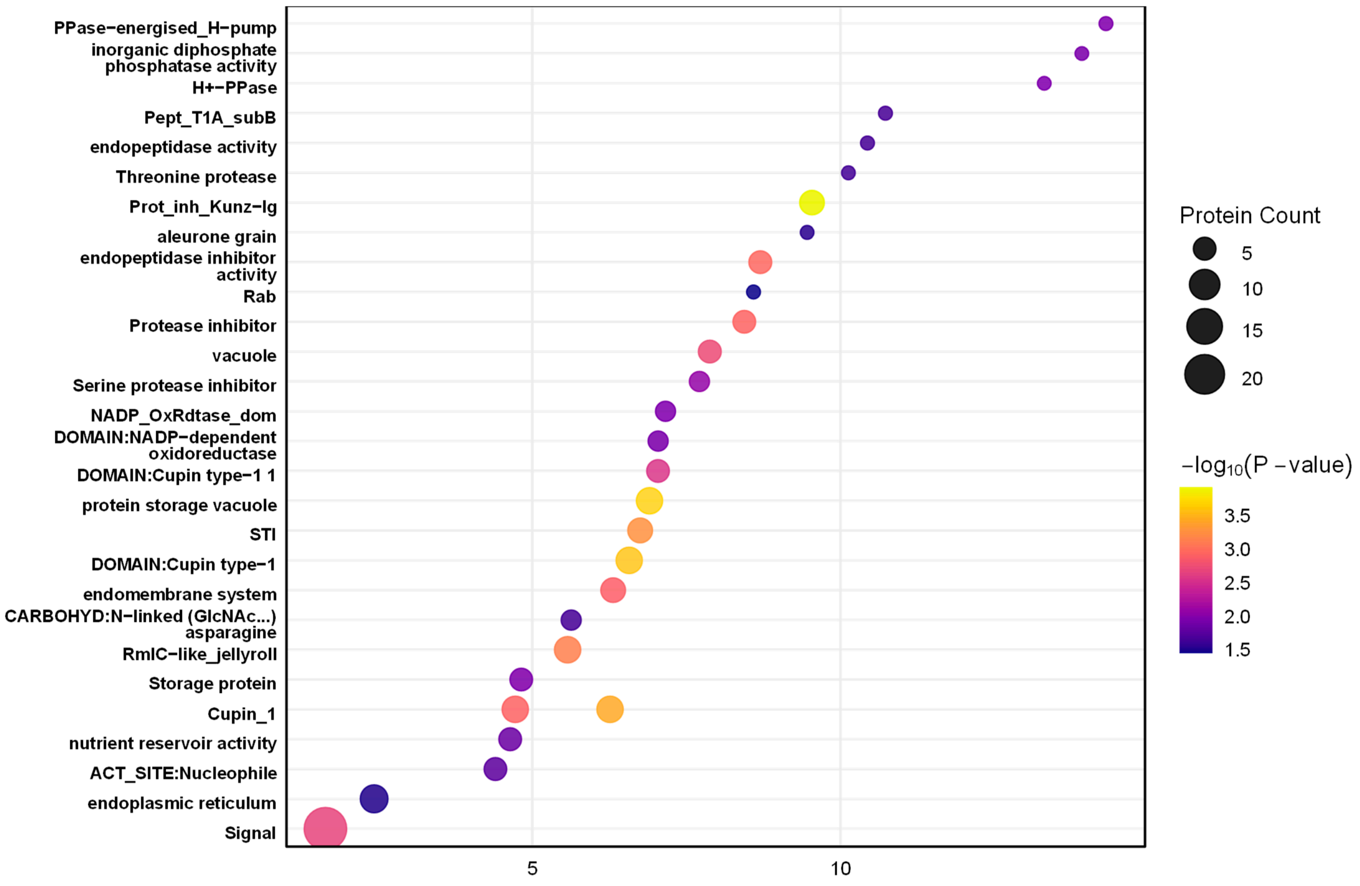

Fold Enrichment

Supplemental Table S1. Differentially expressed proteins identified among E16, Lee2 and HBK-W comparisons.

	**E16 vs Lee2**			
**Gene Identifier**	**Protein ID**	**Accession**	**Log2_Ratio**	**p_value**
GLYMA__20G148300	Beta-conglycinin alpha subunit 1 (CG-alpha-1) (Beta-conglycinin alpha subunit) (allergen Gly m 5)	P0DO16	5.93	4.44E-10
GLYMA__03G213300	Translocator protein homolog	I1JQL0	4.74	3.64E-10
GLYMA_19g34780	Glycinin G3 (Glycinin 11S G3) (Glycinin A1bB2) (allergen Gly m 6) [Cleaved into: Glycinin A1b subunit (Glycinin A subunit); Glycinin B2 subunit (Glycinin B subunit)]	P11828	4.37	2.22E-08
NA	NA	I1L9B1	4.15	1.24E-09
GLYMA__15G055500	dihydropyrimidinase (EC 3.5.2.2)	I1MDY5	3.76	2.55E-07
GLYMA__09G158700	Bowman-Birk serine protease inhibitors family domain-containing protein	K7LE73	3.67	6.25E-05
GLYMA__16G204600	phosphopyruvate hydratase (EC 4.2.1.11)	I1MQ89	3.26	4.58E-06
NA	NA	I1LCM3	3.19	5.53E-05
GLYMA__11G209200	Uncharacterized protein	C6SWA9	3.03	3.67E-06
PARP3 PM38	Protein ADP-ribosyltransferase PARP3 (EC 2.4.2.-) (NAD(+) ADP-ribosyltransferase 3) (ADPRT-3) (Poly [ADP-ribose] polymerase 3) (PARP-3) (Poly[ADP-ribose] synthase 3)	Q9SWB4	2.97	3.40E-06
GLYMA__11G247600	Glyceraldehyde-3-phosphate dehydrogenase (EC 1.2.1.-)	C6TD56	2.84	9.45E-08
GLYMA__04G191500	MI domain-containing protein	I1JXE8	2.68	3.90E-05
GLYMA__13G267900	Uncharacterized protein	A0A0R0GUI6	2.42	9.66E-06
NA	NA	A0A0R0JI98	2.39	7.56E-06
GLYMA__10G124800	Probable voltage-gated potassium channel subunit beta (K(+) channel subunit beta)	I1LAJ3	2.32	2.84E-06
GLYMA__08G342300	Trypsin inhibitor 26 kDa isoform	C6K8D0	2.3	1.14E-06
GGH_SOYBN	Gamma-glutamyl hydrolase (EC 3.4.19.9) (Conjugase) (GH) (Gamma-Glu-X carboxypeptidase)	P93164	2.29	6.34E-07
GLYMA__10G018200	60S ribosomal protein L22-2	C6SVK2	2.26	4.98E-05
GLYMA__15G045100	Carbamoyl phosphate synthase arginine-specific large chain, chloroplastic (EC 6.3.4.16) (EC 6.3.5.5) (Ammonium-dependent carbamoyl phosphate synthetase) (Arginine-specific carbamoyl phosphate synthetase, ammonia chain) (Glutamine-dependent carbamoyl phosphate synthetase)	K7M9L4	2.23	1.05E-04
GLYMA__15G263500	Succinate-semialdehyde dehydrogenase (EC 1.2.1.24)	A0A0R4J5H1	2.21	1.81E-06
GLYMA__18G057700	Malonyltransferase	I1MZV4	2.17	2.79E-06
GLYMA__09G155500	Kunitz trypsin protease inhibitor	B1ACD5	2.15	7.00E-06
GLYMA__19G189400	Uncharacterized protein	I1NAH1	2.13	8.67E-07
GLYMA__19G177200	glutathione transferase (EC 2.5.1.18)	A0A0R0ENV4	2.11	2.40E-06
GLYMA__11G184100	Poly [ADP-ribose] polymerase (PARP) (EC 2.4.2.-)	K7LQ69	2.05	3.73E-06
GLYMA__20G148200	Beta-conglycinin beta subunit 2 (CG-beta-2) (allergen Gly m 5)	F7J077	2.05	7.28E-05
GLYMA__10G062200	Glycosyltransferase (EC 2.4.1.-)	I1L932	2.02	2.19E-05
GLYMA__06G180000	Cell division cycle protein 48 homolog	I1KCD7	1.94	1.25E-06

GLYMA__10G155300	Uncharacterized protein	A0A0R0I4F6	1.8	1.78E-06
GLYMA__15G115300	Cystatin domain-containing protein	A0A0R4J598	1.74	5.01E-05
GLYMA__18G009700	Glyceraldehyde-3-phosphate dehydrogenase (EC 1.2.1.-)	C6TN36	1.68	7.28E-06
GLYMA__20G027200	Nodulin-related protein 1	I1NDJ4	1.67	3.31E-05
GLYMA__13G088000	Fibronectin type III-like domain-containing protein	I1LVZ8	1.58	6.66E-05
GLYMA__12G036700	NADP-dependent oxidoreductase domain-containing protein	C6TKQ3	1.5	1.34E-05
NA	NA	I1L6T4	1.47	9.75E-05
NA	NA	I1N8P5	1.43	9.29E-05
GLYMA__16G037900	Uncharacterized protein	I1MKY3	1.38	2.28E-06
NA	NA	K7MVA9	1.33	9.50E-06
GLYMA__03G190500	phosphopyruvate hydratase (EC 4.2.1.11)	I1JPW5	1.13	2.16E-05
GLYMA__06G319700	Disease resistance protein/LRR protein-related protein	C6ZS00	1.11	3.90E-05
GLYMA__06G078500	Proteasome subunit beta	I1K964	1.07	3.51E-05
GLYMA__19G106800	Glyceraldehyde-3-phosphate dehydrogenase (EC 1.2.1.-)	I1N843	1.04	5.15E-05
GLYMA__02G076600	Seed maturation protein PM31	Q9XET1	-1.11	3.13E-05
GLYMA__11G005800	Cysteine synthase (EC 2.5.1.47)	Q8W1A0	-1.15	3.36E-05
GLYMA__13G035200	alcohol dehydrogenase (EC 1.1.1.1)	A0A0R4J4U4	-1.16	4.73E-05
GLYMA__U032500	Uricase (EC 1.7.3.3) (Urate oxidase)	A0A0R4J455	-1.17	6.94E-05
GLYMA__03G144400	Late embryosis abundant group 4 protein PM9 (Maturation protein) (Soybean seed maturation polypeptides)	Q01527	-1.19	2.09E-05
GLYMA__02G145700	Cupin type-1 domain-containing protein	I1JF86	-1.2	3.22E-05
GLYMA__06G260200	NADP-dependent oxidoreductase domain-containing protein	I1KEA0	-1.26	9.10E-05
KTI1 GLYMA__01G095000	Uncharacterized protein	C6SWW4	-1.27	6.33E-05
GLYMA__08G019300	Reticulon-like protein	C6TGW1	-1.41	4.74E-05
NA	NA	C6T699	-1.42	5.36E-06
GLYMA__15G206800	GH18 domain-containing protein	I1MI18	-1.45	6.18E-05
GLYMA__12G002900	Eukaryotic translation initiation factor 3 subunit I (eIF3i)	C6TL41	-1.49	8.36E-05
GLYMA__06G162500	Ribonuclease	I1KBV2	-1.59	2.85E-05
GLYMA__13G036800	Uncharacterized protein	I1LXB2	-1.66	9.09E-06
GLYMA__13G053000	UDP-arabinopyranose mutase (EC 5.4.99.30)	I1LWV7	-1.89	8.22E-06
GLYMA__15G102600	30S ribosomal protein S8, chloroplastic	K7MAP3	-1.91	6.04E-07
GLYMA__08G341500v4	Kunitz trypsin inhibitor (Protease inhibitor) (Trypsin inhibitor subtype A)	Q39898	-1.95	2.35E-06
GLYMA__13G045800	40S ribosomal protein S7	C6T2H2	-2.02	3.36E-05
GLYMA__15G217700	EF-hand domain-containing protein	I1MIA8	-2.24	2.15E-05
GLYMA__03G190100	Uncharacterized protein	K7KFX8	-2.28	3.43E-05
GLYMA__07G062600	Reticulon-like protein	I1KI22	-2.5	8.50E-05
NA	NA	I1LPI1	-2.56	2.67E-06
GLYMA__02G299000 GLYMA__14G014800	Proteasome subunit beta	I1JJI4	-2.66	1.02E-05
GLYMA__17G127100	26S proteasome regulatory subunit RPN7	A0A0R4J5N5	-2.81	7.85E-06
GLYMA__17G108500	H(+)-exporting diphosphatase (EC 7.1.3.1)	K7ML14	-2.85	1.35E-05
NA	NA	A0A0R0JPG6	-3.04	3.70E-05
NA	NA	Q9FQE7	-3.28	1.71E-07
GLYMA__14G063800	SHSP domain-containing protein	A0A0R0GA14	-3.66	2.22E-07
GLYMA__15G158400	60S ribosomal protein L27	I1MGV6	-3.71	3.34E-07

HSP22.0	22.0 kDa class IV heat shock protein	P30236	-3.73	6.21E-07
GLYMA__08G341000	Uncharacterized protein	I1KYW3	-3.89	1.33E-09
GLYMA__01G177000	Cupin type-1 domain-containing protein	K7K4G2	-4.32	6.59E-08
GLYMA__13G194300	Albumin I chain a domain-containing protein	A0A0R0GQS9	-4.74	5.27E-09
HPSE_SOYBN	Hydrophobic seed protein (HPS) (allergen Gly m 1)	P24337	-4.85	6.73E-10
GLYMA__18G062900	Peptidyl-prolyl cis-trans isomerase (PPIase) (EC 5.2.1.8)	C6SV97	-4.95	1.34E-08
SBP	Sucrose-binding protein (SBP)	Q04672	-6.54	7.09E-11
ITRB_SOYBN	Trypsin inhibitor B (Kunitz-type trypsin inhibitor B)	P01071	-7.32	1.81E-10
	**E16 vs HBK-WF**			
**Gene Identifier**	**Protein ID**	**Accession**	**Log2_Ratio**	**p_value**
GLYMA__20G148300	Beta-conglycinin alpha subunit 1 (CG-alpha-1) (Beta-conglycinin alpha subunit) (allergen Gly m 5)	P0DO16	5.83	5.19E-10
GLYMA__03G213300	Translocator protein homolog	I1JQL0	5.06	2.03E-10
GLYMA_19g34780	Glycinin G3 (Glycinin 11S G3) (Glycinin A1bB2) (allergen Gly m 6) [Cleaved into: Glycinin A1b subunit (Glycinin A subunit); Glycinin B2 subunit (Glycinin B subunit)]	P11828	4.93	7.58E-09
NA	NA	I1L9B1	4.4	7.40E-10
GLYMA__20G130000	Plant basic secretory protein (BSP) family protein	I1NFY4	3.23	3.03E-05
PARP3 PM38	Protein ADP-ribosyltransferase PARP3 (EC 2.4.2.-) (NAD(+) ADP-ribosyltransferase 3) (ADPRT-3) (Poly [ADP-ribose] polymerase 3) (PARP-3) (Poly[ADP-ribose] synthase 3)	Q9SWB4	3	3.12E-06
GLYMA__19G177200	glutathione transferase (EC 2.5.1.18)	A0A0R0ENV4	2.69	2.99E-07
NA	NA	A0A0R0JI98	2.63	3.43E-06
NA	NA	I1LCM3	2.43	4.22E-04
GLYMA__10G124800	Probable voltage-gated potassium channel subunit beta (K(+) channel subunit beta)	I1LAJ3	2.33	2.74E-06
GLYMA__10G018200	60S ribosomal protein L22-2	C6SVK2	2.26	5.10E-05
GLYMA__11G184100	Poly [ADP-ribose] polymerase (PARP) (EC 2.4.2.-)	K7LQ69	2.25	1.71E-06
GLYMA__05G161600	glutathione transferase (EC 2.5.1.18)	A0A0B5E8V1	2.2	5.78E-05
GLYMA_03g07470.1	Protein SLE2 (Em protein) (Protein Glycine max physiologically mature 11) (Protein GmD-19) (Soybean group-1 late embryogenesis abundant protein 2) (Soybean group-1 lea protein 2) (Sle2)	I1JLC8	2.15	1.96E-05
GLYMA__04G191500	MI domain-containing protein	I1JXE8	2.1	2.43E-04
GLYMA__13G267900	Uncharacterized protein	A0A0R0GUI6	2.09	3.11E-05
GLYMA__09G178100	Beta-glucosidase	A0A0R0IIZ3	1.99	4.82E-07
NA	NA	K7MVA9	1.93	4.05E-07
GGH_SOYBN	Gamma-glutamyl hydrolase (EC 3.4.19.9) (Conjugase) (GH) (Gamma-Glu-X carboxypeptidase)	P93164	1.85	3.78E-06
GLYMA__03G002400	Eukaryotic translation initiation factor 3 subunit B (eIF3b) (eIF-3-eta) (eIF3 p110)	I1JK05	1.82	9.90E-05
GLYMA__10G155300	Uncharacterized protein	A0A0R0I4F6	1.73	2.47E-06
GLYMA__06G319700	Disease resistance protein/LRR protein-related protein	C6ZS00	1.69	1.24E-06
GLYMA__13G238200	Eukaryotic translation initiation factor 3 subunit D	I1M229	1.67	2.69E-04
GLYMA__06G054700	SHSP domain-containing protein	I1K8F1	1.65	2.54E-05

GLYMA__20G027200	Nodulin-related protein 1	I1NDJ4	1.64	3.80E-05
GLYMA_01g29480	Protein SLE3 (Soybean group-1 late embryogenesis abundant protein 3) (Soybean group-1 lea protein 3) (Sle3)	C6T0L2	1.63	5.65E-05
GLYMA__10G062200	Glycosyltransferase (EC 2.4.1.-)	I1L932	1.53	1.87E-04
GLYMA__09G151900	Embryo-specific protein ATS3B	I1L3J0	1.47	1.61E-04
GLYMA__09G173200	Glutamine synthetase (EC 6.3.1.2)	I1L432	1.41	3.05E-04
GLYMA__02G262500	Ferritin (EC 1.16.3.1)	I1JIE1	1.33	3.96E-04
GLYMA__17G030200	G.max mRNA from stress-induced gene (H4) (Stress-induced protein H4)	Q43453	1.28	1.50E-05
GLYMA__11G130300	Lipoxygenase (EC 1.13.11.-)	K7LPI4	1.27	1.17E-05
CAT4	Catalase-4 (EC 1.11.1.6)	O48561	1.26	2.82E-05
GLYMA__06G283900	Seed biotin-containing protein SBP65	A0A0R0JWF4	1.26	1.58E-04
GLYMA__15G151000	Sucrose synthase (EC 2.4.1.13)	A0A0R0G9R0	1.25	4.23E-05
GLYMA__08G019400	AAA+ ATPase domain-containing protein	K7L4G7	1.25	2.32E-04
GLYMA__08G175200	Glutathione S-transferase (EC 2.5.1.18)	Q9FQE8	1.19	7.56E-05
GLYMA__14G156400	alcohol dehydrogenase (EC 1.1.1.1)	I1MA91	1.18	1.37E-04
GLYMA__16G189900	Low-temperature-induced 65 kDa protein	K7MID0	1.18	2.12E-04
GLYMA__20G098400	Calreticulin	I1NF03	1.17	2.22E-04
GLYMA__16G037900	Uncharacterized protein	I1MKY3	1.16	1.02E-05
GLYMA__06G144400	40S ribosomal protein S26	C6SXR4	1.13	1.98E-04
GLYMA__12G064300	Osmotin-like protein	I1LQR8	1.1	2.35E-04
GLYMA__06G180000	Cell division cycle protein 48 homolog	I1KCD7	1.09	1.30E-04
GLYMA__18G208000	NADP-dependent oxidoreductase domain-containing protein	I1N334	1.07	4.82E-04
GLYMA_17g03600	Probable bifunctional TENA-E protein (EC 3.5.1.-) (EC 3.5.99.2) (Aminopyrimidine aminohydrolase) (Formylaminopyrimidine amidohydrolase) (Formylaminopyrimidine deformylase) (Seed maturation protein PM36)	Q9SWB6	1.03	1.16E-04
NA	NA	I1LP44	-1.01	2.08E-04
GLYMA__16G050800	Translocon-associated protein subunit beta (TRAP-beta) (Signal sequence receptor subunit beta)	I1MLC3	-1.03	9.41E-05
GLYMA__03G171100	Uncharacterized protein	I1JPC5	-1.05	2.06E-04
GLYMA__13G189500	Cysteine proteinase inhibitor	I1M0K3	-1.07	5.87E-05
GLYMA__13G073200	Translation elongation factor EF1B beta/delta subunit guanine nucleotide exchange domain-containing protein	I1LWB8	-1.1	2.41E-04
GLYMA__13G053000	UDP-arabinopyranose mutase (EC 5.4.99.30)	I1LWV7	-1.15	3.77E-04
GLYMA__20G243700	Formate--tetrahydrofolate ligase (EC 6.3.4.3) (10-formyletrahydrofolate synthetase) (Formyltetrahydrofolate synthetase)	I1NJ85	-1.16	1.56E-04
GLYMA__05G157300	Tubulin alpha chain	I1K3X9	-1.19	3.79E-05
GLYMA__13G291300	Ketol-acid reductoisomerase (EC 1.1.1.86) (Acetohydroxy-acid reductoisomerase) (Alpha-keto-beta-hydroxylacyl reductoisomerase)	I1M3M4	-1.21	4.68E-05
GLYMA__18G299100	Uncharacterized protein	I1N5E4	-1.22	6.50E-05
GLYMA__13G094900	Xyloglucan endotransglucosylase/hydrolase (EC 2.4.1.207)	A0A0R0GKM 0	-1.22	1.32E-04
GLYMA__04G029400 GLYMA__06G029400	AAA+ ATPase domain-containing protein	A0A0R4J305	-1.28	3.28E-04

GLYMA__07G162900	Allene oxide synthase, chloroplastic-like	I1KKQ1	-1.28	3.42E-04
GLYMA__08G232400	EF-hand domain-containing protein	K7L8B5	-1.33	5.90E-05
GLYMA__08G342300	Trypsin inhibitor 26 kDa isoform	C6K8D0	-1.34	9.09E-05
GLYMA__07G254600	Glycosyltransferase (EC 2.4.1.-)	I1KN68	-1.34	1.97E-04
GLYMA__04G012300	Isoflavone reductase homolog 2	Q9SDZ0	-1.38	5.63E-06
GLYMA__06G162500	Ribonuclease	I1KBV2	-1.41	7.20E-05
GLYMA__20G190900	Coatomer subunit alpha	I1NHR3	-1.42	1.92E-04
GLYMA__11G211500	Casein kinase substrate phosphoprotein PP28 domain-containing protein	C6TGZ5	-1.5	3.16E-05
GLYMA__13G214600	Acyl carrier protein	I1M1B6	-1.68	1.70E-05
GLYMA__04G202900	Calnexin homolog	A0A0R0KAL0	-1.7	9.43E-06
GLYMA__13G036800	Uncharacterized protein	I1LXB2	-1.79	4.90E-06
GLYMA__20G146200	Beta-conglycinin beta subunit 1 (CG-beta-1) (Beta-conglycinin beta subunit) (allergen Gly m 5)	P25974	-2.05	2.88E-05
GLYMA__07G062600	Reticulon-like protein	I1KI22	-2.27	1.77E-04
GLYMA__01G032500	threonine--tRNA ligase (EC 6.1.1.3) (Threonyl-tRNA synthetase)	I1J5B4	-2.43	5.51E-05
NA	NA	Q9FQE7	-2.98	3.99E-07
KTI2	Kunitz-type trypsin inhibitor KTI2	P25273	-3.6	2.71E-08
NA	NA	A0A0R0JPG6	-3.63	8.75E-06
HPSE_SOYBN	Hydrophobic seed protein (HPS) (allergen Gly m 1)	P24337	-3.67	8.08E-09
GLYMA__10G207100	Cysteine proteinase	Q43448	-3.9	6.03E-09
GLYMA__15G158400	60S ribosomal protein L27	I1MGV6	-4.14	1.30E-07
GLYMA__13G194300	Albumin I chain a domain-containing protein	A0A0R0GQS9	-5.78	8.92E-10
	**HBK-WF vs Lee2**			
**Gene Identifier**	**Protein ID**	**Accession**	**Log2_Ratio**	**p_value**
KTI2	Kunitz-type trypsin inhibitor KTI2	P25273	3.97	1.15E-08
GLYMA__16G204600	phosphopyruvate hydratase (EC 4.2.1.11)	I1MQ89	3.95	9.01E-07
GLYMA__08G342300	Trypsin inhibitor 26 kDa isoform	C6K8D0	3.64	2.08E-08
GLYMA__11G247600	Glyceraldehyde-3-phosphate dehydrogenase (EC 1.2.1.-)	C6TD56	3.47	1.65E-08
GLYMA__01G032500	threonine--tRNA ligase (EC 6.1.1.3) (Threonyl-tRNA synthetase)	I1J5B4	3.31	4.63E-06
GLYMA__10G207100	Cysteine proteinase	Q43448	3.23	3.23E-08
GLYMA__11G209200	Uncharacterized protein	C6SWA9	3.2	2.35E-06
GLYMA__20G146200	Beta-conglycinin beta subunit 1 (CG-beta-1) (Beta-conglycinin beta subunit) (allergen Gly m 5)	P25974	3.15	8.03E-07
GLYMA__15G055500	dihydropyrimidinase (EC 3.5.2.2)	I1MDY5	2.82	2.97E-06
GLYMA__15G115300	Cystatin domain-containing protein	A0A0R4J598	2.58	2.00E-06
GLYMA__15G045100	Carbamoyl phosphate synthase arginine-specific large chain, chloroplastic (EC 6.3.4.16) (EC 6.3.5.5) (Ammonium-dependent carbamoyl phosphate synthetase) (Arginine-specific carbamoyl phosphate synthetase, ammonia chain) (Glutamine-dependent carbamoyl phosphate synthetase)	K7M9L4	2.56	3.54E-05
GLYMA__15G263500	Succinate-semialdehyde dehydrogenase (EC 1.2.1.24)	A0A0R4J5H1	2.31	1.21E-06
GLYMA__20G148200	Beta-conglycinin beta subunit 2 (CG-beta-2) (allergen Gly m 5)	F7J077	2.21	4.14E-05
GLYMA__02G222400	Fructose-bisphosphate aldolase (EC 4.1.2.13)	I1JH86	1.94	4.12E-06

GLYMA__18G009700	Glyceraldehyde-3-phosphate dehydrogenase (EC 1.2.1.-)	C6TN36	1.76	4.77E-06
GLYMA__17G216000	Aspartate aminotransferase (EC 2.6.1.1)	A0A0R0FQN0	1.67	8.64E-05
GLYMA__08G232400	EF-hand domain-containing protein	K7L8B5	1.63	1.21E-05
GLYMA__13G094900	Xyloglucan endotransglucosylase/hydrolase (EC 2.4.1.207)	A0A0R0GKM 0	1.57	1.78E-05
GLYMA__02G204000	Cytosol aminopeptidase domain-containing protein	I1JGP8	1.52	8.96E-05
GLYMA__13G135500	glutathione transferase (EC 2.5.1.18)	Q9FQ95	1.49	3.70E-05
GLYMA__20G240300	DHAR class glutathione S-transferase	I1NJ59	1.34	1.23E-04
GLYMA__19G189400	Uncharacterized protein	I1NAH1	1.18	1.05E-04
GLYMA__13G291300	Ketol-acid reductoisomerase (EC 1.1.1.86) (Acetohydroxy-acid reductoisomerase) (Alpha-keto-beta-hydroxylacyl reductoisomerase)	I1M3M4	1.16	6.32E-05
GLYMA__13G189500	Cysteine proteinase inhibitor	I1M0K3	1.1	4.64E-05
GLYMA__05G058100	L-lactate dehydrogenase (EC 1.1.1.27)	A0A0R0JRB0	1.03	8.23E-05
GLYMA__09G178100	Beta-glucosidase	A0A0R0IIZ3	-1.06	8.79E-05
GLYMA__10G189900	EF-hand domain-containing protein	A0A0R0HVK7	-1.18	3.87E-05
HPSE_SOYBN	Hydrophobic seed protein (HPS) (allergen Gly m 1)	P24337	-1.18	1.15E-04
GLYMA__08G071300	Protein EXORDIUM-like 2	I1KR17	-1.19	1.19E-04
KTI1 GLYMA__01G095000	Uncharacterized protein	C6SWW4	-1.2	9.71E-05
CAT4	Catalase-4 (EC 1.11.1.6)	O48561	-1.22	3.55E-05
GLYMA__17G030200	G.max mRNA from stress-induced gene (H4) (Stress-induced protein H4)	Q43453	-1.26	1.65E-05
GLYMA__10G243700	NADP-dependent oxidoreductase domain-containing protein	I1LE01	-1.33	8.55E-05
GLYMA__03G144400	Late embryosis abundant group 4 protein PM9 (Maturation protein) (Soybean seed maturation polypeptides)	Q01527	-1.34	7.73E-06
GLYMA__08G259500	NADP-dependent oxidoreductase domain-containing protein	I1KWT3	-1.45	8.52E-05
GLYMA__06G283900	Seed biotin-containing protein SBP65	A0A0R0JWF4	-1.46	5.28E-05
GLYMA_17g03600	Probable bifunctional TENA-E protein (EC 3.5.1.-) (EC 3.5.99.2) (Aminopyrimidine aminohydrolase) (Formylaminopyrimidine amidohydrolase) (Formylaminopyrimidine deformylase) (Seed maturation protein PM36)	Q9SWB6	-1.53	5.15E-06
GLYMA_01g29480	Protein SLE3 (Soybean group-1 late embryogenesis abundant protein 3) (Soybean group-1 lea protein 3) (Sle3)	C6T0L2	-1.56	7.81E-05
GLYMA__15G102600	30S ribosomal protein S8, chloroplastic	K7MAP3	-1.63	2.37E-06
GLYMA__11G098700	Peptidyl-prolyl cis-trans isomerase (PPIase) (EC 5.2.1.8)	A0A0R0HEL8	-1.63	4.76E-05
HSP18.5-C	18.5 kDa class I heat shock protein (HSP 18.5)	P05478	-1.66	1.45E-05
GLYMA__01G130200	Non-specific lipid-transfer protein	I1J7M1	-1.67	4.97E-05
GLYMA__13G088700	Annexin	G3E7M9	-1.84	7.92E-06
KTI3 GLYMA__08G341500v4	Kunitz trypsin inhibitor (Protease inhibitor) (Trypsin inhibitor subtype A)	Q39898	-1.87	3.35E-06
GLYMA__13G045800	40S ribosomal protein S7	C6T2H2	-1.93	4.69E-05
GLYMA__04G216900	Alginate lyase 2 domain-containing protein	K7KLJ9	-2.21	2.33E-05
GLYMA__06G054700	SHSP domain-containing protein	I1K8F1	-2.37	1.22E-06
GLYMA__16G185900	LTI65/LTI78 PGEED repeat domain-containing protein	A0A0R0G3R6	-2.37	2.16E-05
NA	NA	I1LPI1	-2.49	3.38E-06
GLYMA__02G299000 GLYMA__14G014800	Proteasome subunit beta	I1JJI4	-2.55	1.40E-05

GLYMA__17G127100	26S proteasome regulatory subunit RPN7	A0A0R4J5N5	-2.8	8.07E-06
GLYMA__08G341000	Uncharacterized protein	I1KYW3	-3.32	5.42E-09
GLYMA__14G063800	SHSP domain-containing protein	A0A0R0GA14	-3.33	4.99E-07
GLYMA__01G177000	Cupin type-1 domain-containing protein	K7K4G2	-4.27	7.30E-08
HSP22.0	22.0 kDa class IV heat shock protein	P30236	-4.46	1.32E-07
GLYMA__18G062900	Peptidyl-prolyl cis-trans isomerase (PPIase) (EC 5.2.1.8)	C6SV97	-4.69	2.15E-08
SBP	Sucrose-binding protein (SBP)	Q04672	-6.47	7.82E-11
ITRB_SOYBN	Trypsin inhibitor B (Kunitz-type trypsin inhibitor B)	P01071	-7.44	1.56E-10
